# The (amide-)N-methylnicotinamide is a microbe-derived NAD^+^ precursor that drives gut stem cell renewal

**DOI:** 10.1126/sciadv.aef2882

**Published:** 2026-11-06

**Authors:** Jiangmeng Han, Jayson M. Antonio, Jagannatham Naidu Bhupana, Garam Choi, Jiaxing Yang, Eric Chiles, Yan Tong, Mohamed Asik Rajmohamed, Hung-Ting Wu, Dahui Wang, Vik Meadows, Nicolas Lapaque, Jean-Pierre Etchegaray, Rafal Filip, Ta-Chiang Liu, Pawel R. Kiela, Nicholas Bessman, Ronaldo P. Ferraris, Ching-On Wong, Xiaoyang Su, Nan Gao

**Affiliations:** ^1^ Department of Biological Sciences, Rutgers University, Newark, New Jersey, USA.; ^2^ Department of Pharmacology, Physiology, and Neuroscience, Rutgers Biomedical and Health Sciences, Newark, New Jersey, USA.; ^3^ Center for Immunity and Inflammation, Rutgers Biomedical and Health Sciences, Newark, NJ, USA.; ^4^ Department of Medicine, Rutgers Biomedical and Health Sciences, Newark, NJ, USA.; ^5^ Metabolomics Shared Resource, Rutgers Cancer Institute, New Brunswick, NJ 08901, USA.; ^6^ Department of Pathology and Immunology, Washington University in St. Louis, School of Medicine, St Louis, Missouri.; ^7^ Université Paris-Saclay, INRAE, MetaGenoPolis, MGP, 78350 Jouy-en-Josas, France.; ^8^ Université Paris-Saclay, INRAE, AgroParisTech, Micalis Institute, 78350 Jouy-en-Josas, France.; ^9^ Institute of Medicine, Medical College of Rzeszow University, Department of Gastroenterology with IBD Unit, Clinical Hospital, Rzeszow, Poland.; ^10^ Department of Pediatrics, Steele Children’s Research Center, University of Arizona Health Sciences Center, 1501 N. Campbell Ave., Tucson, AZ 85724, USA.; ^11^ Department of Medicine, Robert Wood Johnson Medical School, Rutgers University, New Brunswick, NJ, USA.

## Abstract

Nicotinamide Adenine Dinucleotide (NAD) is a critical cellular coenzyme whose decline associates with various chronic and inflammatory diseases. Improved NAD restoration may reduce disease risks and extend healthy lifespan. We report here that certain commensal bacteria including specific probiotic species produce (amide-)N-methylnicotinamide (N-MNA), which is a structural isomer of methylnicotinamide, a nicotinamide degradative metabolite. Patients with gut dysbiosis and inflammation show reduced stool N-MNA. Stable isotope tracing revealed that N-MNA can be rapidly taken up and incorporated into NAD redox reactions by intestinal epithelial cells (IECs) and non-IECs of fruit fly, mouse, and human. Genetic and pharmacological analyses suggest that N-MNA is converted to NAD^+^ via the Preiss-Handler pathway, independent of the salvage pathway. Cell-type-specific ablation or overexpression of SIRT1, an NAD-consuming enzyme, in *Drosophila*, along with intestinal stem cell (ISC) lineage tracing in mice revealed that systemic N-MNA supplementation promotes ISC regeneration and gut epithelial repair. Compared to known NAD precursors, N-MNA brings a methyl group into host cells, representing a class of microbe-derived NAD precursor that stimulates the tissue regenerative power.

## INTRODUCTION

Nicotinamide Adenine Dinucleotide (NAD) is a coenzyme for sirtuins, RNA polymerases, ribosyl transferases, and PARPs that plays fundamental roles in cellular energy metabolism, DNA repair, and signal transduction (*1*, *2*). NAD constantly cycles between its oxidized form, NAD^+^, and the reduced form, NADH, through the classic redox reaction. In these reactions, NAD^+^ functions as a hydride acceptor, shuttling electrons and a proton among biomolecules to power cellular processes. NAD can be produced through the de novo pathway from the amino acid tryptophan, a process that is active in the liver but insufficient in other tissues due to their lack of key enzymes for de novo synthesis. Recycling of nicotinamide (NAM) via nicotinamide phosphoribosyltransferase (NAMPT) (*3*) is by far the dominant salvage route, and nicotinamide riboside (NR), and nicotinamide mononucleotide (NMN) make comparatively minor contributions under most physiological conditions (*4*). The salvage pathway requires NAMPT and NMN adenylyltransferases (NMNAT1–3), key enzymes that sequentially convert NAM to NMN and to NAD (*5*) (Fig. 1A). Dietary NR can also be converted to NMN that is then processed into NAD (*6*). In addition, nicotinic acid (NA) from the diet or microbial sources can also be processed into NAD via the Preiss-Handler pathway (Fig. 1A). NAD-consuming enzymes, such as sirtuins and poly (ADP-ribose) polymerases (PARPs), break down NAD into NAM and ADP-ribose (*1*, *2*). While the majority of NAM is recycled for NAD^+^ biosynthesis through the salvage pathway, excess NAM is methylated at the pyridine ring nitrogen by nicotinamide N-methyltransferase (NNMT) into N1-methylnicotinamide (N1-MNA) (*7*). N1-MNA cannot be converted back to NAM, and is subsequently oxidized into N1-methyl-2-pyridone-5-carboxamide (2PY) and N1-methyl-4-pyridone-3-carboxamide (4-PY) (*8*, *9*) (Fig. 1A), which are excreted by the kidney (*10*). The tight coupling between NAD biosynthesis and metabolism ensures a stable and sufficient NAD supply (*11*).

**Fig. 1.**
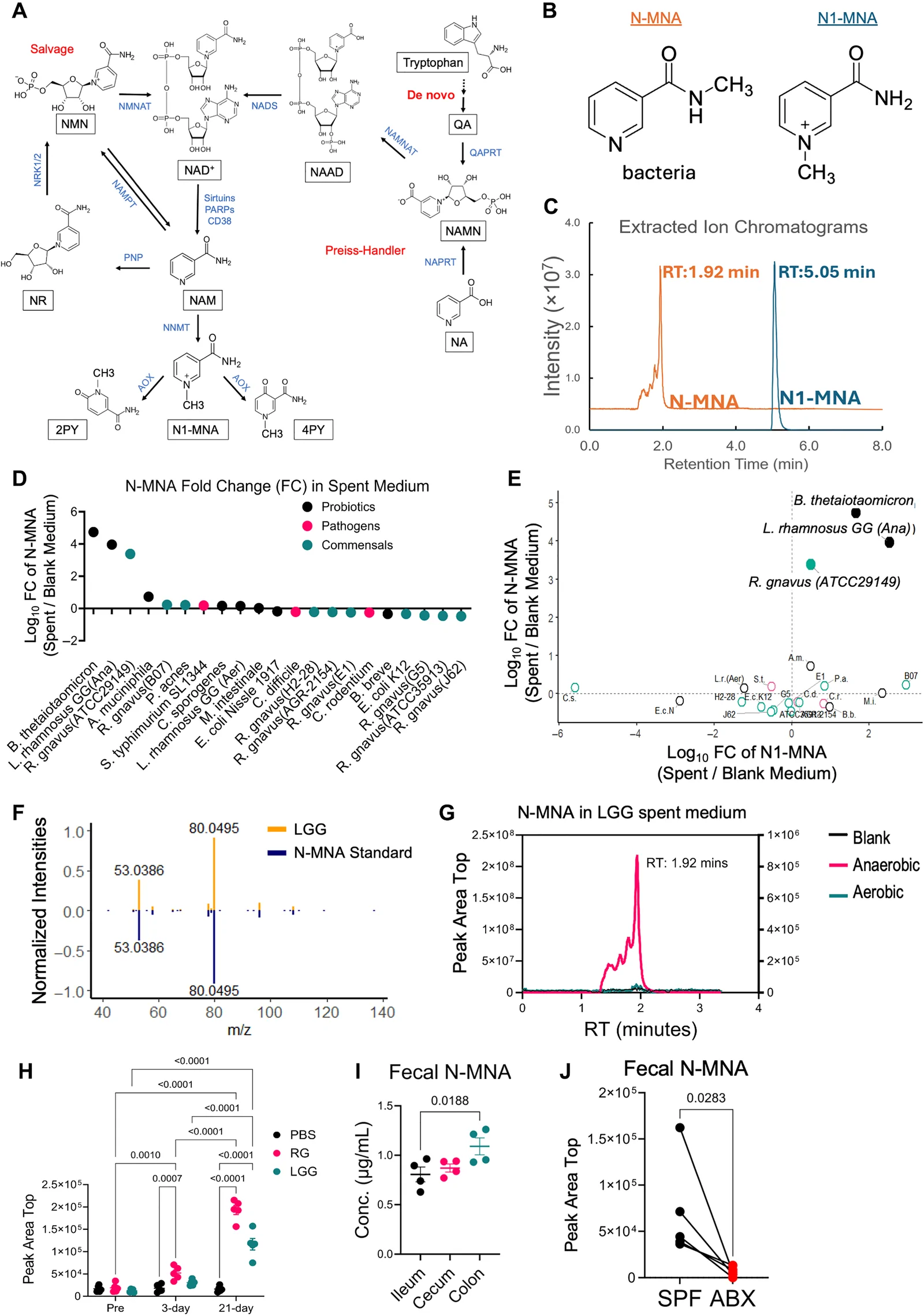
N-MNA is produced by specific commensal bacterial species. (**A**) NAD biosynthetic and metabolic pathways in mammalian cells. The three major pathways are labeled in red and enzymes in blue. (**B**) Chemical structures of N1-MNA, the methylated product of nicotinamide in mammalian cells (left), and its structural isomer N-MNA (right). (**C**) LC-MS profiles of N-MNA (1.92 min) and N1-MNA (5.05 min). (**D**) Twenty bacterial strains were grown to stationary phase. N-MNA production in spent and blank media was measured by LC-MS and plotted as Log_10_ fold-change (FC) of spent over blank medium. Bacteria with probiotic features, commensals, and pathogens are shown as black, green, and pink dots, respectively. (**E**) Correlation plot of N-MNA (*y*-axis) and N1-MNA (*x*-axis) production, shown as Log_10_FC in spent over blank medium. *BT, L*GG, and *RG* (ATCC 29149) are the highest producers of N-MNA and N1-MNA. Dotted lines represent FC = 1. (**F**) MS/MS validation of the 1.92-min peak (N-MNA) from anaerobic *L*GG spent medium (yellow) by comparison with standard N-MNA (blue). (**G**) LC-MS profiles of N-MNA detected in anaerobic *Lactobacillus rhamnosus* GG spent medium (red, left *y*-axis), blank medium (black), and aerobic spent medium (green, right *y*-axis). (**H**) Germ-free mice were inoculated with PBS (black), RG (red), or LGG (green). Feces were collected pre-inoculation and 3 and 21 days after mono-colonization for LC-MS detection of N-MNA (n = 5 per group). Two-way ANOVA with Tukey’s multiple-comparison test. (**I**) Ileal, cecal, and colonic luminal contents from adult SPF mice (n = 4) were spiked with ^2^H_4_-N-MNA and analyzed by LC-MS. Ratios of ^2^H_4_-N-MNA to unlabeled N-MNA peak areas were used to calculate N-MNA concentrations. One-way ANOVA with Fisher’s LSD test; ileum versus colon. (**J**) LC-MS detection of fecal N-MNA in SPF mice before and after 7-day antibiotic treatment (n = 5). Paired *t* test.

Cellular NAD levels significantly decline during natural aging, and NAD content is also reduced under various pathologies that affect gut, brain, peripheral nerves, skeletal muscle, kidney, or cardiovascular systems (*12*–*14*). Thus, there is a constant interest in NAD restoration strategies or compounds to extend healthspan and reduce disease risk (*15*). Impaired NAD metabolism has been strongly associated with the risk and pathogenesis of inflammatory bowel diseases (IBD) (*16*, *17*). NAD^+^ depletion compromises the barrier integrity and mitochondrial functions, contributing to the disease severity (*18*). As barrier disruption is linked to microbial dysbiosis and inflammatory activities, the shifts in host–microbe interactions in IBD patients may further accelerate NAD^+^ consumption and depletion (*19*). Thus, a vicious cycle of NAD^+^ depletion, barrier disruption, and inflammation may exacerbate disease progression. On the other hand, supplementation of canonical NAD^+^ precursors such as NAM, NA, NMN, and NR has been shown to restore barrier function and suppress inflammation in preclinical IBD models (*20*, *21*).

Bacterial metabolites are central players in maintaining intestinal homeostasis (*22*), and gut bacteria capable of NAD synthesis have been investigated in IBD recurrence or resolution (*23*). However, due to its large molecular weight and electric charge, extracellular NAD^+^ is unable to diffuse across cell membranes on its own. Certain gut microbes are known to synthesize NAM and NA (collectively referred to as niacin). These microbially made NAD precursors can be taken up through various transporters on the gut epithelial cells and utilized for host cell NAD biosynthesis (*24*). To what extent the commensal gut microbiota contributes to the host NAD turnover, stability, and function remains to be fully elucidated (*25*).

Here, we report the discovery of a microbiota-derived NAD precursor, (amide-)N-methyl-nicotinamide. It is a structural isomer of N1-methyl-nicotinamide with its methyl group on the amide nitrogen, which is produced by certain commensal bacteria. By using stable isotope-labeled tracer, we demonstrate the uptake and metabolic conversion of N-methyl-nicotinamide into NAD^+^ in human gut epithelial cells, mouse organoids, and *Drosophila melanogaster*. Genetic and pharmacological experiments show revealed that (amide-)N-methyl-nicotinamide is rapidly incorporated into NAD redox reaction through the Preiss-Handler pathway. In the gut, (amide-)N-methyl-nicotinamide robustly promotes stem cell renewal, tissue regeneration, and host survivability through the NAD^+^-dependent deacetylase SIRT1. Humans who have severe gut dysbiosis and inflammation have reduced fecal (amide-)N-methyl-nicotinamide levels. These data reveal a previously unknown microbial NAD precursor that may be leveraged for systemic NAD^+^ restoration and disease intervention.

## RESULTS

### Identification of a bacterium-derived methylnicotinamide isomer

Nicotinamide (NAM) is a major byproduct of NAD^+^ consumption and the excessive NAM is methylated by nicotinamide N-methyltransferase (NNMT) at its pyridine nitrogen. NNMT is a mammalian enzyme absent in lower invertebrates such as the fruit fly (*26*). The product of this methylation reaction is N1-methyl-nicotinamide (CAS 3106-60-3), whose carbon of the methyl group attached to the ring nitrogen is designated as “N1” (Fig. 1A). We will refer to it as N1-MNA hereafter. We and others have reported that N1-MNA has anti-inflammatory and vaso-protective effects in endothelial and gut mucosal tissues (*27*, *28*).

In addition, our liquid chromatography–mass spectrometry (LC-MS) analysis chromatographically resolved a structural isomer of N1-MNA (Fig. 1, B and C): (amide-)N-methylnicotinamide (CAS 114–33-0), whose methyl-group is attached to the amide nitrogen (Fig. 1B). We refer to (amide-)N-methylnicotinamide as N-MNA hereafter. Compared to N1-MNA that is widely detected as a nicotinamide degradative metabolite in approximately 900 human cell lines (*29*), N-MNA was found or described in the human metabolomic encyclopedia dataset (*30*). Amide nitrogen is generally a poor nucleophile and a methyltransferase capable of its methylation has not been reported in eukaryotic cells.

To investigate the non-mammalian cellular source of N-MNA, we performed LC-MS analysis of representative human gut bacterial members of the dominant phyla (*31*), including *Bacteroidetes* (*Bacteroides thetaiotaomicron, Muribaculum intestinale*), *Firmicutes* (*Lactobacillus rhamnosus GG, Ruminococcus gnavus*)*,* and *Proteobacteria* (*Escherichia coli K12* and *Nissle 1917*). We also tested members of minor phyla such as *Actinomycetota (Bifidobacterium breve, Propionibacterium acnes)* and *Verrucomicrobia* (*Akkermansia muciniphila*). These bacterial species were selected based on their reported probiotic properties, e.g., *L. rhamnosus GG* (*LGG*), *B. breve*, *A. muciniphila, and E. coli Nissle 1917* (*32*–*37*), or associations with metabolic and inflammatory diseases, e.g., *R. gnavus*, *M. intestinale, P. acnes* (*38*–*42*). We also included enteric pathogens: *Clostridioides difficile*, *Salmonella typhimurium*, and *Citobacter rodentium*, known to cause intestinal inflammation (*43*–*46*). All bacterial species were cultured in their specific growth medium to the stationary phase. LC-MS was performed on the blank and spent medium of each bacterial culture. Analysis of fold changes of N-MNA in spent medium over blank medium (Fig. 1, D and E), or net intensity of N-MNA in the spent medium (fig. S1A), uncovered that *B. thetaiotaomicron, L. rhamnosus GG* (*ATCC53103*)*, and R. gnavus* (*ATCC 29149*) were the highest N-MNA producers. These bacteria also produced N1-MNA (Fig. 1E, fig. S1, B and C). The other bacteria, including the pathogens, produced negligible N-MNA (Fig. 1, D and E). The chemical identities of the N-MNA (Fig. 1F) and N1-MNA peaks (fig. S1, D and E) were validated by tandem mass spectrometry (MS/MS) analyses.

Most of our tested bacteria are obligate anaerobes. *LGG* can be cultured under both aerobic and anaerobic conditions. Interestingly, anaerobically cultured *LGG* produced abundant N-MNA at a three-order-of-magnitude over the aerobically cultured LGG (Fig. 1G). This is interesting as it suggests that the aerobic condition, often resulting from severe gut inflammation (*47*), may suppress N-MNA production by facultative anaerobes. In addition to MNAs, NA, NR, and NAM are highly produced by *B. thetaiotaomicron*, *P. acnes, and R. gnavus (ATCC29149),* respectively (fig. S1F). In contrast, N-MNA production is highly restricted to the limited commensal or probiotic species.

To test if the bacterial producers of N-MNA may produce this metabolite in vivo, we mono-associated germ-free (GF) mice with *LGG* or *R. gnavus* (ATCC 29149). Fecal samples were collected at pre-colonization (i.e., the GF condition), and 3 and 21 days after association. LC-MS revealed a significant increase in fecal N-MNA and N1-MNA from *R. gnavus* or *LGG* mono-colonized mice (Fig. 1H, fig. S1G).

We then performed quantitative LC-MS and determined the absolute intestinal luminal N-MNA abundances in adult specific pathogen-free (SPF) C57BL/6 mice. The colonic lumen contains approximately 1.09 μg/mL N-MNA, while the ileum has 0.81 μg/mL (Fig. 1I, fig. S1H). We then tested the impact of microbial dysbiosis on fecal N-MNA in SPF mice by administrating a broad-spectrum antibiotic cocktail (ABX) through drinking water (*48*). This led to a markedly reduced fecal N-MNA (Fig. 1J) and N1-MNA (fig. S1I), but not NAM (fig. S1J). Thus, the microbe-derived N-MNA may represent a metabolic product from the “healthy” commensal gut microbiota.

### Human gut inflammation and dysbiosis are associated with reduced stool N-MNA

We next investigated if N-MNA can be detected in human stool samples. As gut inflammation and dysbiosis form a vicious cycle during the IBD pathogenesis (*49*), we were also interested to determine whether there might be an association between the stool N-MNA and gut dysbiosis or inflammatory conditions. We extracted stool metabolites and resolved them by LC-MS from 25 ulcerative colitis (UC), 29 Crohn’s disease (CD) patients, and 20 healthy control (HC) donors. CD patients showed an overall lower stool N-MNA than healthy controls (Fig. 2A), while N1-MNA exhibited no difference among subject groups (fig. S2A).

**Fig. 2.**
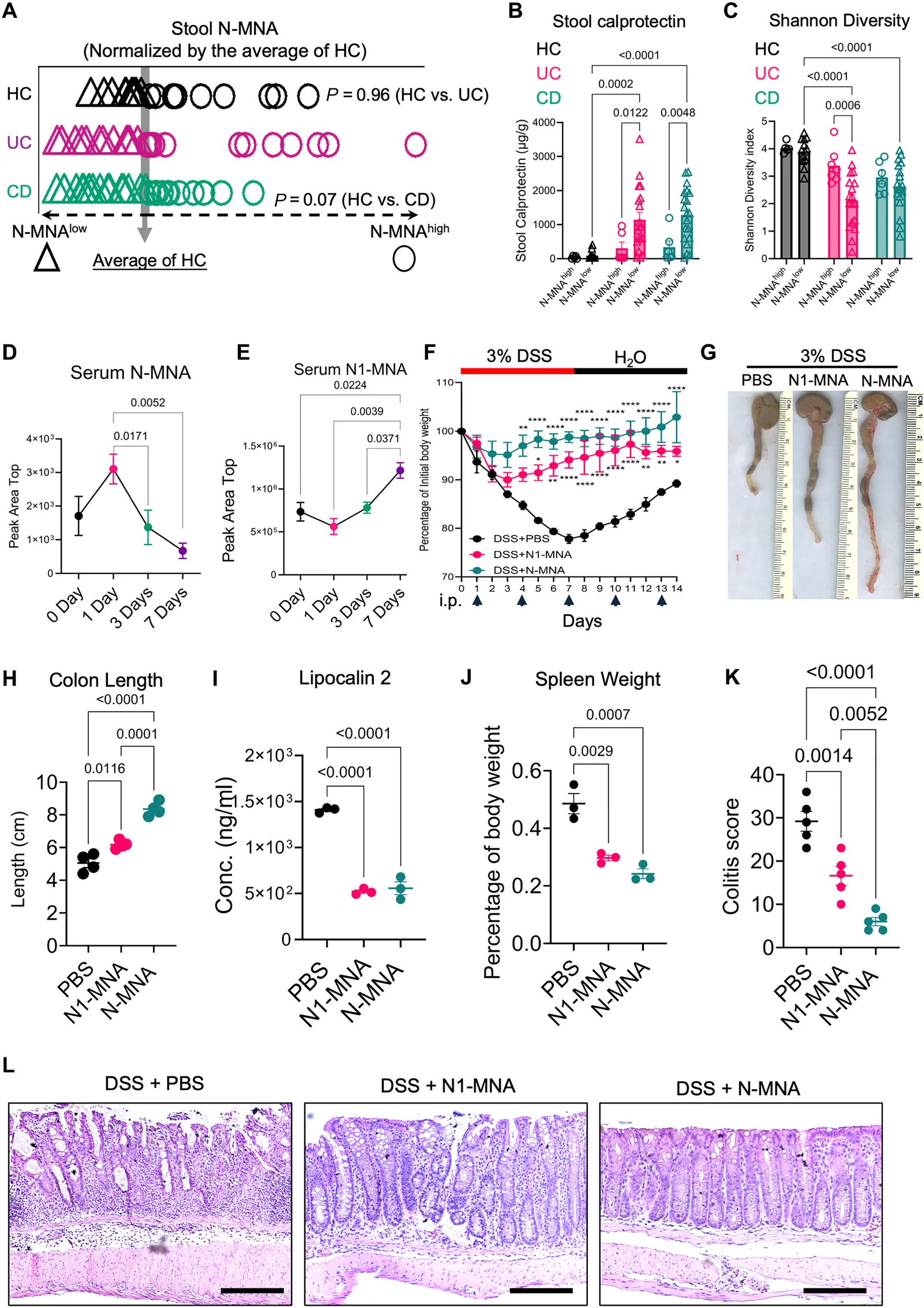
N-MNA protects against murine colitis and inversely associates with gut inflammation and dysbiosis in IBD patients. (**A**) Stool N-MNA was measured by LC-MS from 25 ulcerative colitis (UC, pink), 29 Crohn’s disease (CD, green), and 20 healthy control (HC, black) donors. Samples were ordered by increasing N-MNA abundance normalized to the HC average (gray line); values above or below the HC average are shown as circles or triangles. One-way ANOVA with Fisher’s LSD test. (**B**) Stool calprotectin concentrations in HC, UC, and CD cohorts comparing individuals with high or low stool N-MNA (N-MNA^high^ and N-MNA^low^). Two-way ANOVA with Tukey’s multiple-comparison test. (**C**) Bacterial 16S rRNA sequencing of the above groups. Shannon diversity was calculated for individual samples and compared between N-MNA^high^ and N-MNA^low^ individuals within HC, UC, and CD cohorts. Two-way ANOVA. (**D**) Adult C57BL/6 mice received 3% DSS in drinking water. Serum was collected before treatment (day 0) and on days 1, 3, and 7. Serum N-MNA was measured by LC-MS (n = 3 per time point). One-way ANOVA. (**E**) Serum N1-MNA from the above experiment was measured by LC-MS (n = 3 per time point). One-way ANOVA. (**F**) Adult C57BL/6 mice received 3% DSS for 7 days followed by water for 7 days. Mice received i.p. N-MNA (30 mg/kg, green), N1-MNA (30 mg/kg, red), or PBS (black) on days 1, 4, 7, 10, and 13 (n = 4 per group). Two-way ANOVA. (**G**) Representative colons collected on day 14. (**H**) Colon lengths (n = 4 per group); one-way ANOVA. (**I**) Cecal fecal lipocalin-2 measured by ELISA (n = 3 per group); one-way ANOVA. (**J**) Spleen weight as percentage of body weight (n = 3 per group). (**K**) Colitis histopathological scores for mucosal architecture, epithelial damage, inflammatory infiltration, and crypt abscesses (n = 5 per group). (**L**) Representative day-14 colon histology. Scale bar, 50 μm.

Stool calprotectin is a clinical biomarker of intestinal inflammation, with its concentration at 50–120 μg/g and > 120 μg/g representing moderate and severe inflammatory activities (*50*). Although UC and CD patients had higher average stool calprotectin than healthy controls, a huge variation was observed likely reflecting different disease states of patients (fig. S2B). To examine potential correlation of stool N-MNA with inflammation, we used the healthy control’s mean stool N-MNA value to segregate IBD patients into stool N-MNA^high^ and N-MNA^low^ groups, respectively. Interestingly, stool N-MNA^low^ patients showed a significantly higher calprotectin than N-MNA^high^ patients in both UC (*P* = 0.0122) and CD (*P* = 0.0048) patients (Fig. 2B). No correlation was found in healthy controls (Fig. 2B). Examination of stool N1-MNA showed a similar correlation pattern with calprotectin (fig. S2C).

We next analyzed the correlation between stool N-MNA and gut dysbiosis, using the Shannon Diversity Index (H), a key ecological measure of microbial biodiversity reflecting microbial richness and evenness. A lower Shannon index is indicative of a more severe gut dysbiosis condition (*51*). UC patients of lower stool N-MNA had a significantly lower Shannon Diversity than stool N-MNA^high^ patients (Fig. 2C), supporting a correlation between low stool N-MNA with severe dysbiosis. No correlation was observed for N1-MNA and Shannon Diversity (fig. S2D), likely reflecting the reality that human host cells can also produce N1-MNA.

### N-MNA protects against experimental colitis in mice

We then used a model of dextran sodium sulfate (DSS) experimental colitis to model gut inflammation and dysbiosis in vivo (*52*, *53*). Adult wild-type (WT) SPF C57BL/6 mice were administered 3% DSS in drinking water to induce the disease. Serum samples were collected from mice before and after DSS challenge. LC-MS analysis revealed that serum N-MNA declined significantly on day 7 compared to the disease onset at day 1 (Fig. 2D). Serum N1-MNA increased on day 7 compared to day 1 (Fig. 2E), suggesting a different metabolic flux of these two metabolic isomers.

To investigate whether N-MNA supplementation may modify DSS colitis pathogenesis or recovery, adult WT SPF mice were administered 3% DSS in drinking water for 7 days to induce colitis and switched to regular water for 7 days to allow recovery (Fig. 2F). Mice were intraperitoneally injected with N-MNA (30 mg/kg body weight) or N1-MNA (30 mg/kg body weight) every 3 days throughout the experiment (arrows, Fig. 2F). This dosage was based on published intraperitoneal delivery dose of NA (*21*), NR (NCT05561738), NAM (NCT07156929), NMN (*20*), or NAD (*54*) in mice or human clinical trials. Compared to PBS-treated mice that exhibited progressive body weight loss up to ∼80% in response to DSS (Fig. 2F), N-MNA-treated mice showed only a 5% body weight loss during the first 3 days (Fig. 2F). DSS colitis is often associated with colon shortening (*55*, *56*). This protection by N-MNA appeared greater than N1-MNA previously shown to be anti-colitogenic (*28*), as postmortem analysis revealed a significantly longer colon in N-MNA-treated mice (Fig. 2, G and H). The ameliorated disease in N-MNA treated mice was further supported by their significantly reduced fecal lipocalin 2 level (*57*) (Fig. 2I), spleen/body weight ratio (Fig. 2J), and the colitis score (Fig. 2, K and L).

### N-MNA is taken up by gut epithelial cells and incorporated into the NAD redox reaction

To determine if N-MNA can be taken up by gut epithelial cells, we administrated stable isotope deuterium (^2^H_4_) labeled N-MNA, ^2^H_4_-N-MNA, to the human colon epithelial Caco-2 cells (Fig. 3A). At 15 mins, 30 mins, 1, 3, 6, 12 and 24 hours after addition of ^2^H_4_-N-MNA, cells were washed by PBS to eliminate the un-incorporated tracer. Cell metabolites were extracted and resolved by LC-MS. ^2^H_4_-N-MNA was rapidly taken up by cells with an intracellular ^2^H_4_-N-MNA peak detected at 3 hours (Fig. 3B). ^2^H_4_-N-MNA declined afterwards for up to 12 hours and increased again at 24 hours, indicating an active catabolism (Fig. 3B). Mirroring the uptake of ^2^H_4_-N-MNA was a surge of intracellular ^2^H_3_-NAD^+^ that peaked at 3 hours and stabilized at 24 hours (Fig. 3C).

**Fig. 3.**
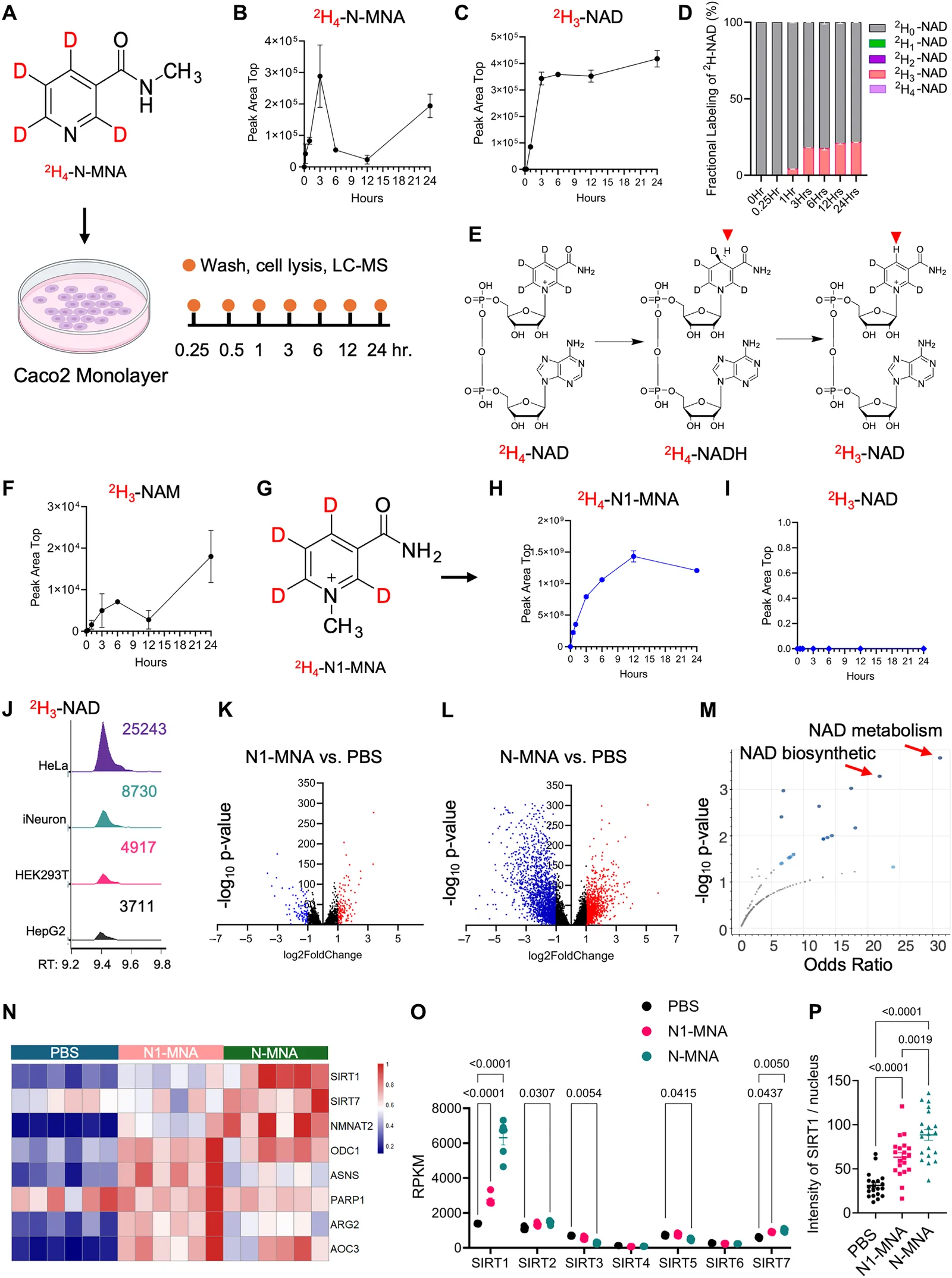
N-MNA is converted to NAD^+^ in human cells and regulates the NAD related transcriptome. (**A**) ^2^H_4_-N-MNA was added to confluent Caco2-BBE cells at 60 μM. Cells were harvested at 0 (pre-treatment), 0.25, 0.5, 1, 3, 6, 12, and 24 hours after extensive washing with cold PBS to remove unincorporated tracer (n = 3 per time point). Metabolites were extracted for LC-MS analysis. Created in BioRender. Han, J. (2026) https://BioRender.com/v0o3sfe. (**B** and **C**) LC-MS determination of intracellular (B) ^2^H_4_-N-MNA and (C) ^2^H_3_-NAD following ^2^H_4_-N-MNA addition during time course. (**D**) Fractional intracellular NAD labeling: ^2^H_0_-NAD (gray), ^2^H_1_-NAD (green), ^2^H_2_-NAD (purple), ^2^H_3_-NAD (pink), and ^2^H_4_-NAD (indigo). (**E**) Schematic of ^2^H_4_-N-MNA incorporation into ^2^H_4_-NAD and conversion to ^2^H_3_-NAD through NAD-NADH redox cycling (red arrowhead). (**F**) LC-MS determination of intracellular ^2^H_3_-NAD following ^2^H_3_-NAM addition during time course. (**G**) Caco2-BBE cells were treated with ^2^H_4_-N1-MNA as described in (A). (**H** and **I**) LC-MS determination of intracellular (H) ^2^H_4_-N1-MNA and (I) ^2^H_3_-NAD following ^2^H_4_-N1-MNA addition during time course. (**J**) Intracellular ^2^H_3_-NAD measured 16 hours after 60 μM ^2^H_4_-N-MNA supplementation in HeLa, human iNeuron, HEK293T, and HepG2 cells (n = 3 for HeLa, HEK293T, and HepG2; n = 1 for iNeuron). (**K** and **L**) Bulk RNA-seq was performed on Caco2-BBE cells treated with PBS, N1-MNA, or N-MNA for 24 hours (n = 6 per condition). Volcano plots show significantly up- and downregulated transcripts following (K) N1-MNA or (L) N-MNA treatment versus PBS. Transcripts with >2-fold increases or decreases are shown in red or blue, respectively. (**M**) Enrichr pathway analysis identified NAD metabolism and NAD biosynthesis (red-arrow) as the most significantly affected pathways by N-MNA and N1-MNA. (**N**) Heatmap of KRPM values for the top changed NAD metabolism and biosynthesis genes among the three groups. (**O**) Seven SIRT genes in PBS-, N1-MNA-, or N-MNA-treated cells (n = 6 per group); two-way ANOVA. (**P**) Nuclear SIRT1 signals after PBS, N-MNA, or N1-MNA treatment (n = 20 images per condition); ordinary one-way ANOVA.

The ^2^H-labeled NAD^+^ is mostly in the form of ^2^H_3_-NAD (M3) (Fig. 3D), indicating the incorporation of ^2^H_4_-N-MNA into the NAD^+^/NADH redox cycle. At 24 hours, the ^2^H_3_-NAD accounted for 21.8% of total cellular NAD (Fig. 3D). While the incorporation of ^2^H_4_-N-MNA gives rise to [2,4,5,6]-^2^H_4_-NAD, the 4-^2^H on the nicotinamide ring is quickly lost in the NAD-NADH redox cycles (red arrowheads, Fig. 3E) due to the pro-R and pro-S specificity of the various NAD-dependent dehydrogenases (*58*, *59*). As a result, [2,4,5]-^2^H_3_-NAD becomes the major form of labeled NAD.

In mammalian cells, NAD is catabolized to nicotinamide (NAM) by sirtuins, CD38, PARPs, or other NAD-consuming enzymes (*13*). Indeed, ^2^H_3_-NAM was observed to increase at 6 hours and after 12 hours (Fig. 3F), whose temporal change between 12 and 24 hours paralleled the intracellular ^2^H_4_-N-MNA accumulation (Fig. 3B). ^2^H_3_-N1-MNA, the methylated product of ^2^H_3_-NAM, was also detected from 6 hours (fig. S3A). Neither endogenous N-MNA, nor ^2^H_3_-N-MNA, was detected (fig. S3, B and C), suggesting that Caco-2 cells do not produce N-MNA de novo, nor can they re-synthesize N-MNA from ^2^H_3_-NAD.

A trace of intracellular ^2^H_4_-NA was detected in early time point (fig. S3D), but no ^2^H_3_-NA was detected (fig. S3E), reflecting the lack of a mammalian nicotinamidase (*60*) that converts NAM to NA. To determine if an impurity of ^2^H_4_-NA in ^2^H_4_-N-MNA could contribute to the observed ^2^H_3_-NAD, we performed a 48-hour pulse tracing for ^2^H_4_-N-MNA in Caco2 cells, and quantified the absolute ^2^H_4_-NA amount in cell culture medium at 0 hr. (input) by spiking in a known concentration of unlabeled NA. The absolute ^2^H_3_-NAM in 48-hr cell culture medium was also determined by LC-MS via a known medium concentration of unlabeled NAM (fig. S3F). The input ^2^H_4_-NA was less than 0.03% of the produced extracellular ^2^H_3_-NAM. Consistent with continuous intracellular NAD turnover, ^2^H_3_-N1-MNA was also progressively accumulated in the extracellular medium (fig. S3G). These results demonstrate that the quantity of ^2^H_4_-NA impurity in the tracer was insufficient to account for the secreted ^2^H_3_-NAM, which is only one of the metabolites derived from the intracellular ^2^H_3_-NAD turnover. In addition, no metabolic degradation of ^2^H_4_-N-MNA or its conversion to other metabolites (such as ^2^H_4_-NA) was observed in cell-free culture medium over 24 hours (fig. S3, H and I). Thus, ^2^H_4_-N-MNA taken up by Caco2 cells accounts for the observed biosynthesis of the labeled NAD and its derivatives.

In contrast to ^2^H_4-_N-MNA, stable isotope tracing of ^2^H_4_-N1-MNA supplemented at the same concentration showed a rapid uptake without being converted into NAD or any of its derivatives, such as NAM (Fig. 3, G to I, fig. S3J).

To investigate if N-MNA can be used for NAD biosynthesis in other cell types, we performed similar ^2^H_4_-N-MNA tracing experiments in human iPSC-derived neurons (iNeuron), hepatocytes (HepG2), kidney (HEK293), and cervix epithelial cells (HeLa). We detected ^2^H_3_-NAD in all these cell types after ^2^H_4−_N-MNA supplementation (Fig. 3J). Thus, the microbiota-derived N-MNA can be used for NAD^+^ biosynthesis in different host tissues.

### N-MNA activates NAD-SIRT1 signaling

To investigate the biological function of N-MNA, we performed bulk RNA-sequencing analysis on Caco-2 cells treated with N1-MNA or N-MNA for 24 hours. Principal component analysis (PCA) revealed a separation of the transcriptomes of N1-MNA or N-MNA-treated cells from control PBS-treated cells (fig. S4A). N-MNA elicited more robust and extensive transcriptomic changes than N1-MNA (Fig. 3, K and L, fig. S4B). NAD^+^ biosynthetic (*P* = 0.00021) and NAD^+^ metabolism (*P* = 0.00053) were identified by enrichment analysis as the top gene networks in N- or N1-MNA-treated cells (Fig. 3M). The most significantly changed genes by N-MNA include *Sirtuins (SIRTs)* that are NAD-dependent deacetylases (*61*) and the *NMNAT* enzyme that belongs to the NAD salvage pathway (Fig. 3N) (*62*, *63*). Among the 7 human *SIRT*s, *SIRT1* was the most elevated by N-MNA and moderately increased by N1-MNA (Fig. 3O). *PPARGC1* and *FOXO3* are the downstream target genes for SIRT1 (*64*, *65*), and both were significantly elevated (fig. S4C), indicating a N-MNA stimulated SIRT1 signaling program. Immunostaining revealed an increased nuclear SIRT1 in N-MNA-treated cells (Fig. 3P, fig. S4D). Western blot showed that a higher N-MNA concentration increased cellular SIRT1 abundance, but neither N1-MNA nor NAM had a similar effect (fig. S4, E and F).

### NAD biosynthesis from N-MNA is conserved in invertebrates

NAD metabolism and function are highly conserved in living organisms due to their fundamental importance (*66*). *D. melanogaster* has a conserved *SIRT1* ortholog that utilizes NAD^+^ but lacks an orthologous enzyme (mammalian NNMT) that methylates NAM (*26*). Thus, *Drosophila* does not make endogenous N1-MNA. To investigate the metabolic fate of N-MNA in lower animal models, we fed wild-type fruit flies with ^2^H_4_-N-MNA (or ^2^H_4_-N1-MNA) in sucrose solution for 5 hours, followed by feeding with plain water for 30 min to wash out unabsorbed metabolites. The bodies and heads of the flies were separately analyzed by LC-MS (Fig. 4, A to D). ^2^H_4_-N-MNA and ^2^H_4_-N1-MNA were detected in both bodies and heads that were pooled from 29 ∼ 33 flies fed with the tracers (Fig. 4, B and D). The detection of these tracers in the fly heads suggested that they could be taken up by the gut cells and distributed systemically.

**Fig. 4.**
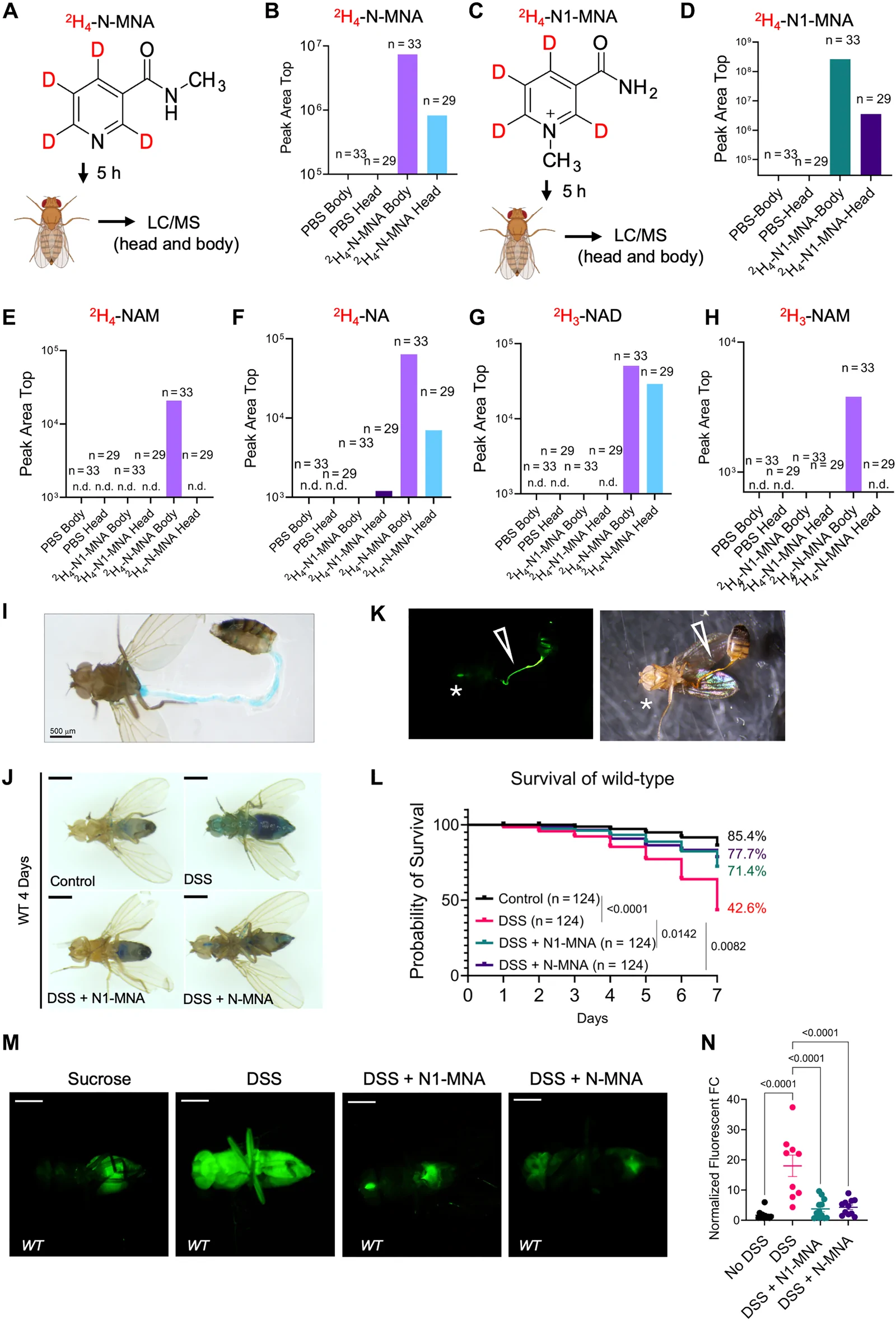
Conserved incorporation of N-MNA into NAD biosynthesis in *Drosophila* protects against gut damage and lethality. (**A**) Adult male WT *Drosophila* were fed ^2^H_4_-N-MNA in water for 5 hours, followed by water for 30 min. Bodies and heads were separated, homogenized, and extracted for LC-MS analysis. Flies fed 0.1% PBS in water served as controls. Created in BioRender. Han, J. (2026) https://BioRender.com/3d2v562. (**B**) LC-MS quantitation of ^2^H_4_-N-MNA in fly bodies and heads. (**C**) Experimental design for ^2^H_4_-N1-MNA tracing using the protocol in (A). (**D**) LC-MS quantitation of ^2^H_4_-N1-MNA in bodies and heads. (**E** to **H**) LC-MS quantitation of (E)^2^H_4_-NAM, (F) ^2^H_4_-NA, (G) ^2^H_3_-NAD, and (H) ^2^H_3_-NAM in bodies and heads of flies fed with PBS, ^2^H_4_-N-MNA, or ^2^H_4_-N1-MNA. (**I**) Representative dissected midgut from a WT flies fed 2.5% EDS diet for 4 days and visualized by light microscopy. (**J**) Adult WT *Drosophila* were fed sucrose solution (control), 5% DSS, 5% DSS containing 100 μM N1-MNA, or 5% DSS containing 100 μM N-MNA for 4 hours daily, followed by 2.5% EDS diet. Representative images were taken on day 4 after the first DSS treatment. Scale bar, 500 μm. (**K**) Representative WT flies fed 2% FSS diet for 4 days and imaged by fluorescence microscopy. White triangles indicate the midgut; asterisk indicates the head. (**L**) Seven-day survival plot of WT *Drosophila* treated with control, 5% DSS, 5% DSS + N1-MNA, or 5% DSS + N-MNA (n = 124 per group). Endpoint survival percentages are shown. Log-rank test. (**M**) Adult WT *Drosophila* received the treatments described in (J) for 4 hours daily, followed by 2% FSS diet. Representative images were taken on day 4. Scale bar, 500 μm. (**N**) Whole-body fluorescence represented as fold-change, normalized to untreated control flies without FSS diet (n = 9–12 per group); One-way ANOVA.

In flies fed with ^2^H_4_-N-MNA, ^2^H_4_-NAM and ^2^H_4_-NA were detected in the bodies (purple bars in Fig. 4, E and F). Only ^2^H_4_-NA but not ^2^H_4_-NAM was found in the heads (blue bar in Fig. 4F). ^2^H_3_-NAD was detected in both bodies and heads (purple and blue bars, Fig. 4G). ^2^H_3_-NAD (M3) accumulated at 18.74% of total NAD in the fly heads and at 8.77% in the fly bodies (fig. S5A). ^2^H_3_-NAM was detected only in the bodies (purple bar, Fig. 4H). Thus, the absorbed ^2^H_4_-N-MNA likely entered the circulation, reached distal tissues such as the brain, and was subsequently used for NAD biosynthesis. In contrast, in flies fed with ^2^H_4_-N1-MNA, neither ^2^H_4_-NAM, ^2^H_4_-NA nor ^2^H_3_-NAD was detected in any parts of the flies (Fig. 4, E to H).

### N-MNA protects against gut damage and lethality via gut stem cell *SIRT1*

NAD is fundamentally important for survival and longevity (*67*). We next investigated if N-MNA is protective in *Drosophila*. Wild-type (WT) flies, when treated with 5% DSS in 5% sucrose solution, displayed an increased gut permeability that was visualized by a colored diet containing 2.5% Erioglaucine disodium salt (EDS) (*68*) (Fig. 4, I and J) in both sexes (fig. S5B) or by a fluorescent diet containing 2% Fluorescein Sodium Salt (FSS) (*69*) (Fig. 4K). In these DSS experiments, the lethality of WT flies typically increases over time (red line in Fig. 4L). Administering WT flies with N-MNA dissolved in the drinking water significantly improved their survivability during the DSS treatment, from 42.6% (red line) to 77.7% (purple line, Fig. 4L). N1-MNA provided a reduced protection at 71.4% (blue line, Fig. 4L). DSS-induced gut leakiness in these groups was quantified by measuring whole-body fluorescence (*70*). Both MNAs reduced gut permeabilities caused by DSS (Fig. 4, M and N).

*Drosophila Sirt1* promotes longevity (*71*); however, its role in gut barrier function has not been investigated. Compared to WT flies, *Sirt1* mutant (*Sirt1^17^/Sirt1^2A-7-11^*) flies showed significantly increased gut leakage at steady state, suggesting that loss of *Sirt1* compromises gut barrier at the baseline, reminiscent of findings in *Sirt1* knockout mice (*72*, *73*). DSS treatment exacerbated the lethality (Fig. 5A) and the gut leakage in these mutant flies (Fig. 5, B and C). In these *Sirt1* mutants, N-MNA and N1-MNA failed to rescue these phenotypes (Fig. 5, A to C), supporting that their beneficial effects are mediated through *Sirt1*.

**Fig. 5.**
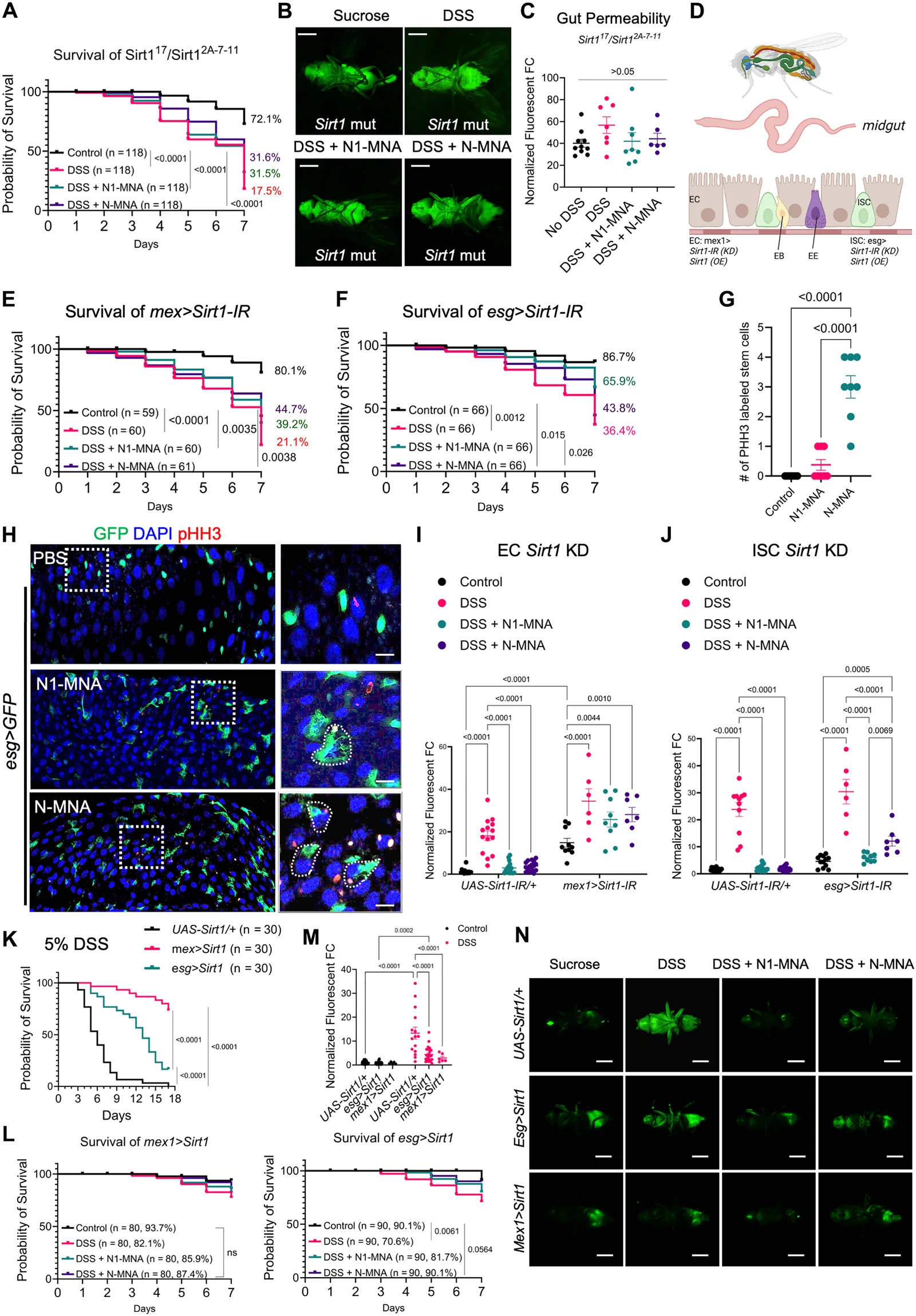
*Drosophila* midgut *Sirt1* mediates N-MNA’s protection. (**A**) Seven-day survival plot of Sirt1 mutant (*Sirt1^I7^/Sirt1^2A-7-11^*) flies receiving control, 5% DSS, DSS + N1-MNA, or DSS + N-MNA (n = 118 per group). Endpoint survival percentages are indicated; log-rank test. (**B**) *Sirt1* mutants received control, 5% DSS, DSS + 100 μM N1-MNA, or DSS + 100 μM N-MNA for 4 hours per day followed by 2% FSS diet. Representative day 4 images. Scale bar, 500 μm. (**C**) Whole-body fluorescence of *Sirt1* mutant *Drosophila*, shown as fold-change normalized to untreated blank flies (n = 10 per treatment). One-way ANOVA. (**D**) Schematic of *Drosophila* midgut cell types. *mex1* and *esg* drivers were used for *Sirt1* knockdown (KD) or overexpression (OE) in epithelial cells and intestinal stem cells (ISCs), respectively. Created in BioRender. Yang, A. (2026) https://BioRender.com/cmumsuv. (**E** to **F**) Seven-day survival plot of (E) *mex > Sirt1-IR* and (F) *esg > Sirt1-IR Drosophila* treated with control (n = 59), 5% DSS (n = 60), DSS + N1-MNA (n = 60), or DSS + N-MNA (n = 61). (**G** and **H**) pHH3^+^ stem cells in *esg > GFP* midguts after control, N1-MNA, or N-MNA treatment (n = 8 per group); one-way ANOVA. (H) Representative pHH3 staining; scale bar, 5 μm. (**I** and **J**) *mex1 > Sirt1-IR* (I)*, esg > Sirt1-IR* (J), and genetic control *(UAS-Sirt1-IR/+) Drosophila* received the treatments described in (B). Whole-body fluorescence normalized to untreated blank flies are shown [n = 6–15 per group in (I); n = 6–16 per group in (J)]; two-way ANOVA. (**K**) Survival plot of *UAS-Sirt1/+* (control, black), *mex1 > Sirt1* (EC-specific OE, pink), and *esg > Sirt1* (ISC-specific OE, green) *Drosophila* fed 5% DSS. Endpoint survival at day 17 is shown; log-rank tests. (**L**) Seven-day survival plot of *mex > Sirt1* and *esg > Sirt1* flies receiving control, DSS, DSS + N1-MNA, or DSS + N-MNA; log-rank tests. (**M** and **N**) Whole-body fluorescence in control, *mex1 > Sirt1*, and *esg > Sirt1* flies receiving control or 5% DSS followed by 2% FSS (n = 6–25 per group); two-way ANOVA. (N) Representative day-4 images; scale bar, 500 μm.

To investigate which gut cell types mediate *Sirt1*’s effects, we knocked down *Sirt1* in the enterocytes (EC) using the *mex1*-*GAL4* driver (*74*) and in intestinal stem cells (ISC) using the *esg*-*GAL4* driver (*75*, *76*) (Fig. 5D), respectively. RNA interference constructs targeting *Sirt1* (*Sirt1-IR*) were used in each experiment. Genetic control individuals (*Sirt1-IR not driven by GAL4*) exhibited a survival rate comparable to WT flies following DSS treatment (43.03% vs.42.57% on day 7) (fig. S5C).

The EC-specific *Sirt1* knockdown (*KD*) flies, in response to DSS treatment, exhibited a reduced survival at 21.09% (Fig. 5E) compared to 43.03% in the genetic control group (fig. S5C). Neither N1-MNA nor N-MNA rescued survival (Fig. 5E). However, in ISC-specific *Sirt1 KD* flies, only N-MNA’s protection was markedly diminished (43.79% vs. 36.43%, *P* > 0.05, Fig. 5F) while N1-MNA still improved survival to 65.92% in DSS-treated flies (p = 0.026, Fig. 5F). Thus, N-MNA’s protective effects are sensitive to the *Sirt1* knockdown in ISCs.

Damage-induced *Drosophila* midgut regeneration occurs via ISC division (*77*). We took advantage of GFP-labeled ISCs in the *esg > GFP* flies to examine the effects of N-MNA on midgut ISC division (*78*). We subjected these flies to 5% DSS with PBS, with N-MNA or N1-MNA treatment. Midgut cell proliferation was then determined by staining for phospho-histone H3 (pHH3), a mitotic cell marker (*79*, *80*). N-MNA treatment markedly increased pHH3^+^GFP^+^ ISC numbers compared to PBS or N1-MNA-treated flies (Fig. 5, G and H). Although N1-MNA increased total pHH3^+^ cell number (fig. S5D), it did not change pHH3^+^GFP^+^ ISC number. Multiple pHH3^+^GFP^−^ epithelial cells were also found to be adjacent to GFP^+^ ISCs in N-MNA-treated flies (Fig. 5H), suggesting that N-MNA not only promoted ISCs’ proliferation, but also their differentiation into other cells. The clustering of these ISCs is reminiscent of injury-induced migratory and proliferative behavior of ISCs (*81*).

We then determined N-MNA’s effects on the barrier functions in these cell-type-specific *Sirt1 KD* flies. *Sirt1* KD in EC is sufficient to increase gut permeability in the absence of DSS, and DSS further exacerbated the gut permeability in these EC-specific *Sirt1* KD flies (Fig. 5I). Neither N-MNA nor N1-MNA rescued the barrier defects in these EC-specific *Sirt1* KD flies (Fig. 5I, fig. S5E).

N-MNA’s rescuing effects on barrier function were significantly blunted by ISC-specific *Sirt1* KD, reducing from 12.5-fold barrier protection in genetic control flies to 2.5-fold protection in ISC-specific *Sirt1 KD* flies (Fig. 5J, fig. S5F). In contrast, N1-MNA still significantly restored barrier integrity (Fig. 5J). Thus, ISC-specific *Sirt1* is partially required for N-MNA–mediated protection.

Furthermore, we generated *esg* > *Sirt1* and *mex1* > *Sirt1* flies to overexpress *Sirt1* in ISCs or ECs, respectively, and examined N-MNA’s effects in these flies. Upon DSS treatment, *mex1* > *Sirt1* flies exhibited a more pronounced rescuing effect than *esg > Sirt1* flies in both survival (red line, Fig. 5K) and permeability (Fig. 5L). Neither N-MNA nor N1-MNA further promoted survival rates or gut barrier functions (Fig. 5, L to N). These results suggested both necessity and sufficiency of *Sirt1* to mediate the protection.

### N-MNA is converted to NAD^+^ via the Preiss-Handler pathway

NAMPT is the rate-limiting enzyme of the salvage pathway for making the vast majority of intracellular NAD (*82*) (Fig. 1A). To test if N-MNA is converted in cells to NAD through this pathway, we grew the *Nampt^fl/fl^;Vil-Cre* (*NamptΔ^IEC^*) and wild type (WT) mouse ileal spheroids enriched for stem cells (*83*) (Fig. 6A). Total NAD^+^ levels in spheroid lysates were determined by NAD fluorometric assay. *NamptΔ^IEC^* spheroids had a NAD^+^ content at 21.6% of the WT spheroids at the baseline (Fig. 6B). We supplemented ^2^H_4_-N-MNA to these spheroids, and the total NAD^+^ abundance was elevated by 10% in *NamptΔ^IEC^* spheroids after 6 hours, while the same supplement increased NAD^+^ by 2.1-fold in WT spheroids (Fig. 6B). LC-MS detected the production of ^2^H_3_-NAD in both WT and *NamptΔ^IEC^* spheroids (Fig. 6, C and D), constituting 26.33% of total NAD in WT spheroids and 33.89% in *NamptΔ^IEC^* spheroids 12 hours after supplementation (fig. S6, A and B).

**Fig. 6.**
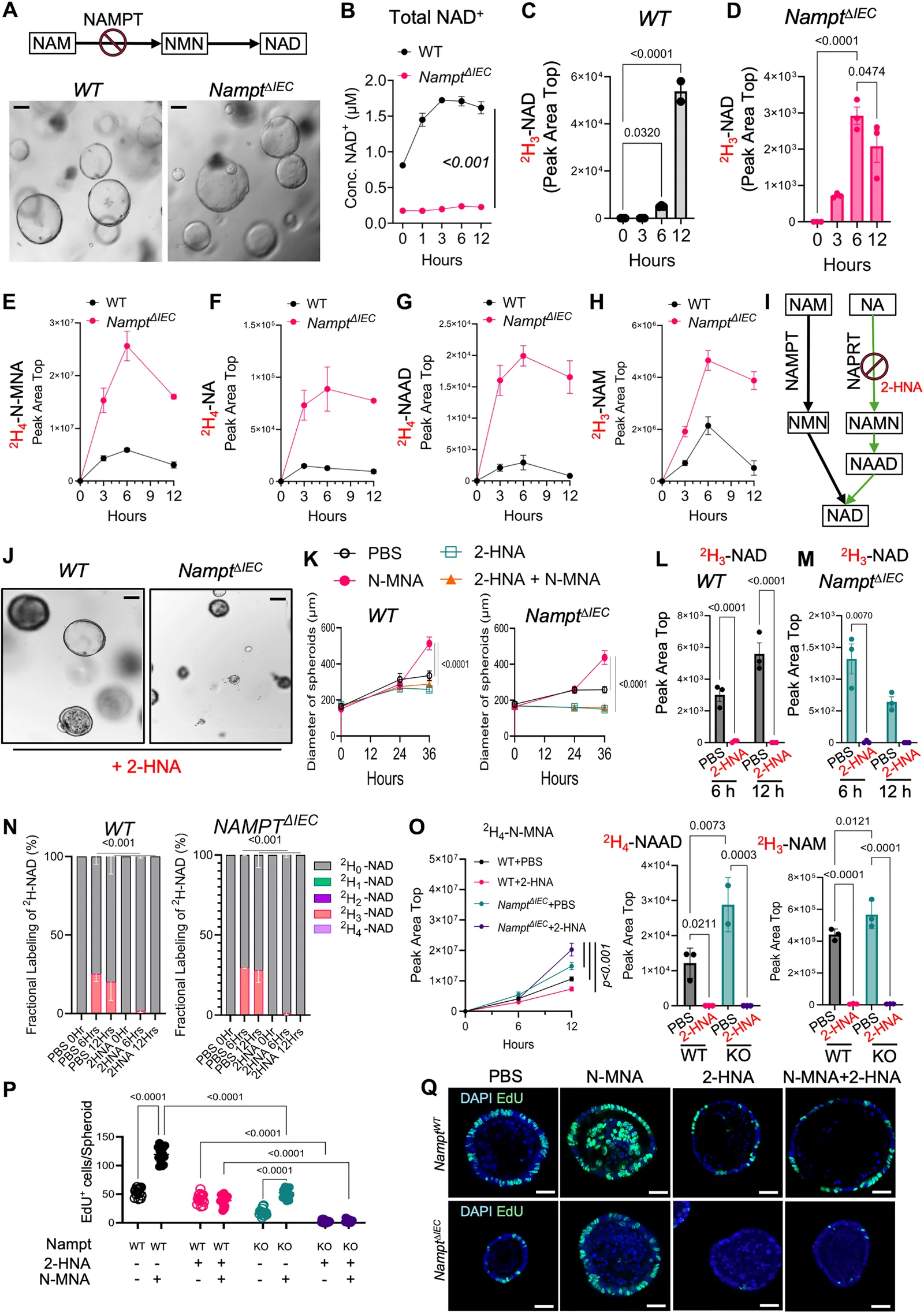
N-MNA is converted to NAD through the Preiss-Handler pathway. (**A**) NAMPT is a rate-limiting salvage-pathway enzyme converting NAM to NMN for NAD biosynthesis. *WT* and *Nampt^fl/fl^;Villin-Cre* (*NamptΔ^IEC^*) ileal crypts were cultured as spheroids. Day-2 images are shown. Scale bar, 100 μm. (**B**) Day-2 *WT* and *NamptΔ^IEC^* spheroids were treated with 60 μM ^2^H_4_-N-MNA and collected at 0, 1, 3, 6, and 12 hours. Total intracellular NAD^+^ was measured fluorometrically and normalized to total protein (n = 3 per-genotype per-time point); two-way ANOVA. (**C** and **D**) LC-MS measurement of ^2^H_3_-NAD in (C) *WT* and (D) *NamptΔ^IEC^* spheroids at 0, 3, 6, and 12 hours; one-way ANOVA. (**E** to **H**) Intracellular (E) ^2^H_4_-N-MNA, (F) ^2^H_4_-NA, (G) ^2^H_4_-NAAD, and (H) ^2^H_3_-NAM from the experiment in (B) (n = 3 per-genotype per-time point). (**I**) NAPRT converts NA to NAMN in the Preiss-Handler pathway; 2-HNA inhibits NAPRT. (**J**) *WT* and *NamptΔ^IEC^* spheroids were treated with 1 mM 2-HNA for 24 hours and imaged after 24 hours. Scale bar, 100 μm. (**K**) Spheroids treated with or without 2-HNA and N-MNA, after 12 hours diameters were measured by ImageJ; two-way ANOVA. (**L** to **N**) *WT* and *NamptΔ^IEC^* spheroids were treated with 1 mM 2-HNA or PBS for 24 hours, ^2^H_4_-N-MNA was added at a final concentration of 60 μM. After washing, spheroids were collected at 6 and 12 hours. (L-M) Intracellular ^2^H_3_-NAD peak area top in *WT* and *NamptΔ^IEC^* spheroids (n = 3). (N) Fractional NAD labeling (^2^H_0_-^2^H_4_) at 0, 6, and 12 hours; two-way ANOVA. (**O**) Intracellular ^2^H_4_-N-MNA at 6 and 12 hours and ^2^H_4_-NAAD and ^2^H_3_-NAM at 12 hours in *WT* and *NamptΔ^IEC^* spheroids (n = 3); two-way ANOVA. (**P** and **Q**) EdU labeling of *WT* and *NamptΔ^IEC^* spheroids treated with 2-HNA or N-MNA. EdU was added 30 min before fixation, and EdU^+^ cells were manually quantified per spheroid; two-way ANOVA. Scale bar, 100 μm.

We analyzed the metabolic fate of ^2^H_4_-N-MNA in *NamptΔ^IEC^* spheroids to elucidate how it was converted to NAD in the absence of NAMPT. First, *NamptΔ^IEC^* spheroids showed an increased uptake of ^2^H_4_-N-MNA compared to WT spheroids (Fig. 6E). Second, the production of ^2^H_4_-NA and ^2^H_4_-NAAD, intermediate metabolites of the Preiss-Handler pathway, was detected in both WT and *NamptΔ^IEC^* spheroids but was more elevated in the latter (Fig. 6, F and G). Third, none of the salvage pathway intermediates (^2^H_4_-NR, ^2^H_4_-NMN, or ^2^H_4_-NAM) was detected in WT or *NamptΔ^IEC^* spheroids (fig. S6, C to E). Instead, the isotope-labeled NAM (Fig. 6H), NR, NMN, and N1-MNA were all ^2^H_3_ species (fig. S6, F and G) derived from consuming ^2^H_3_-NAD^+^. These results suggest that ^2^H_4_-N-MNA is entered into the Preiss-Handler pathway for NAD biosynthesis. The drastically reduced ^2^H_3_-NMN in *NamptΔ^IEC^* spheroids reflected a lack of NAMPT that makes NMN (fig. S6G).

The observed lower ^2^H_3_-NAD in *NamptΔ^IEC^* spheroids (Fig. 6, B to D) reflected an increased consumption of NAD in these cells, consistent with their increased ^2^H_3_-NAM (Fig. 6H). qPCR analysis revealed significantly elevated Preiss-Handler pathway enzyme genes *NADS* and *NAPRT* in *NamptΔ^IEC^* compared to WT spheroids (fig. S6H), suggesting that in the absence of NAMPT-mediated salvage, Preiss-Handler pathway enzymes are increased to compensate.

We then investigated if pharmacological inhibition of the Preiss-Handler pathway would abolish the conversion of N-MNA to NAD. By converting NA to NAMN for NAD synthesis, NAPRT is situated at the entrance of the Preiss-Handler pathway (Fig. 6I). WT and *NamptΔ^IEC^* spheroids were treated with 2-hydroxynicotinic acid (2-HNA, Table1), an inhibitor for nicotinic acid phosphoribosyltransferase (NAPRT) (*84*) (Fig. 6I). After 24 hours of 2-HNA (or PBS) treatment, ^2^H_4_-N-MNA was added to spheroids for stable isotope tracing. 2-HNA-treated *NamptΔ^IEC^* spheroids exhibited visible growth inhibition, reflected by their smaller sizes (Fig. 6, J and K, fig. S6, I and J). 2-HNA significantly blunted the N-MNA-induced growth of both WT and KO spheroids (Fig. 6, J and K).

We analyzed the metabolic fate of ^2^H_4_-N-MNA in spheroids with or without 2-HNA treatments. PBS-treated WT and *NamptΔ^IEC^* spheroids showed the synthesis of ^2^H_3_-NAD; however, WT and *NamptΔ^IEC^* spheroids pre-treated with 2-HNA contained little ^2^H_3_-NAD (Fig. 6, L to N). 2-HNA treatment accumulated ^2^H_4_-N-MNA and blocked the productions of ^2^H_4_-NAAD and ^2^H_3_-NAM in both WT and *NamptΔ^IEC^* spheroids (Fig. 6O), suggesting that 2-HNA inhibited the entry of ^2^H_4_-N-MNA into the Preiss-Handler pathway for NAD synthesis.

As NAD is crucial for stem cell proliferation by fueling energy metabolism in the TCA cycle and glycolysis (*85*), we labeled cycling cells using EdU incorporation in spheroids treated with N-MNA, 2-HNA, or both. While N-MNA alone significantly increased EdU^+^ cells in WT and *NamptΔ^IEC^* spheroids, its effects were abolished by 2-HNA (Fig. 6, P and Q). The overall proliferative activity in N-MNA-supplemented *NamptΔ^IEC^* spheroids remained 2.7 times lower than that of WT spheroids (Fig. 6P). Thus, 2-HNA blocks the conversion of N-MNA to NAD^+^ and reduces N-MNA’s effects on cell proliferation.

We performed similar 2-HNA experiments in Caco2 cells to validate the observed effects in human cells. 2-HNA treatment dramatically reduced the formation of intracellular ^2^H_3_-NAD and ^2^H_3_-NAM accompanied by a marked accumulation of ^2^H_4_-N-MNA and ^2^H_4_-NA (fig. S6, K to N). These results further support that ^2^H_4_-N-MNA is likely metabolized through the Preiss–Handler pathway in both mice and humans.

### Investigating potential host amidase(s) for metabolizing N-MNA

To investigate the potential host enzymes that metabolize N-MNA, we first tested the *Drosophila* nicotinamidase, Naam, which converts NAM into NA (*86*). We focus on Naam as it is the only annotated nicotinamidase in fly and we speculate its involvement in metabolizing N-MNA. By crossing flies carrying the *Naam^102^* and *Naam^123^* alleles respectively, we developed trans-heterozygous *Naam^102/123^* mutant flies. LC-MS revealed that endogenous NA was diminished in *Naam^102/123^* mutants, with an accumulation of NAM, supporting that the Naam function is deficient in these flies (fig. S6O)*.* Stable isotope tracing of ^2^H_4_-N-MNA in *Naam^102/123^* mutants showed an increased uptake of ^2^H_4_-N-MNA and a marked accumulation of ^2^H_4_-NAM compared to the WT flies (fig. 6, P and Q), indicating a potential metabolic pathway from N-MNA to NAM in *Drosophila*. These changes were accompanied by an increased ^2^H_3_-NAM (fig. S6R), concordant to an impeded metabolic flux in the mutant flies due to their reduced NAM-NA convertibility. As ^2^H_3_-NAM was derived from ^2^H_3_-NAD, *Naam-deficient* flies can still metabolize N-MNA into NAD.

Mammals do not have Naam. Carboxylesterase 2 (CES2) (*87*) and arylacetamide deacetylase (AADAC) (*88*) are two amidases enriched in the intestinal epithelial cells. We tested the ability of recombinant CES2 and AADAC in metabolizing ^2^H_4_-N-MNA in vitro. Neither ^2^H_4_-NA nor ^2^H_4_-NAM was produced by these enzymes from ^2^H_4_-N-MNA after 24 hours (not shown). Thus, unknown mammalian enzymes are responsible for metabolizing N-MNA into intermediates that can enter the Preiss-Handler pathway.

### N-MNA drives ISC renewal and gut epithelial repair in mice

The above spheroid studies were done in a stem-cell-enriched condition. We next tested the function of N-MNA in a typical mouse enteroid model containing both the stem cell and differentiated compartment. As NAM was recently reported to be taken up by cells (*24*), we included NAM as a comparison. Enteroids were grown from a Paneth cell reporter mouse line (*Lyz1^3’UTR-IRES-CreER^; Rosa26R^tdTomato^*) that we have reported previously. Paneth cells can be labeled by the tdTomato reporter upon 4-OHT treatment (*89*). Enteroids were treated with various metabolites or PBS. N-MNA most significantly increased the number of EdU^+^ cells (fig. S7, A and B), the number of Paneth cells (fig. S7, C and D), and enlarged the size of the epithelial buds (fig. S7E). NAM did not induce significant changes with the published concentrations (*24*), whereas N1-MNA moderately increased proliferation and Paneth cell numbers.

To investigate if supplementing N-MNA could promote ISC-mediated tissue repair in a disease model, we performed genetic lineage-tracing of Lgr5^+^ ISCs in DSS-challenged mice. *Lgr5^CreER-IRES-EGFP^; Rosa26R^tdTomato^* mice were first i.p. injected with tamoxifen to label ISCs. They were then subjected to colitis induction with 3% DSS treatment for 7 days through the drinking water. Mice were intraperitoneally injected with PBS, N-MNA, or N1-MNA every 3 days throughout the 2-week time course (Fig. 7A). After 7 days of recovery with regular water, the colon tissues were analyzed by immunohistochemistry. ISCs and their progenies were labeled by the tdT. N-MNA-treated mice showed 8 times more tdT^+^ lineage tracing events in distal colons, compared to PBS-treated mice (white arrows indicate red stripes, Fig. 7, A and B). N1-MNA moderately increased ISC lineage tracing (Fig. 7B). We also analyzed the ISC proliferation in the small intestines of these mice using OLFM4 and PCNA as markers. Mice treated with N-MNA contained the greatest number of OLFM4^+^PCNA^+^ ISCs (Fig. 7, C and D).

**Fig. 7.**
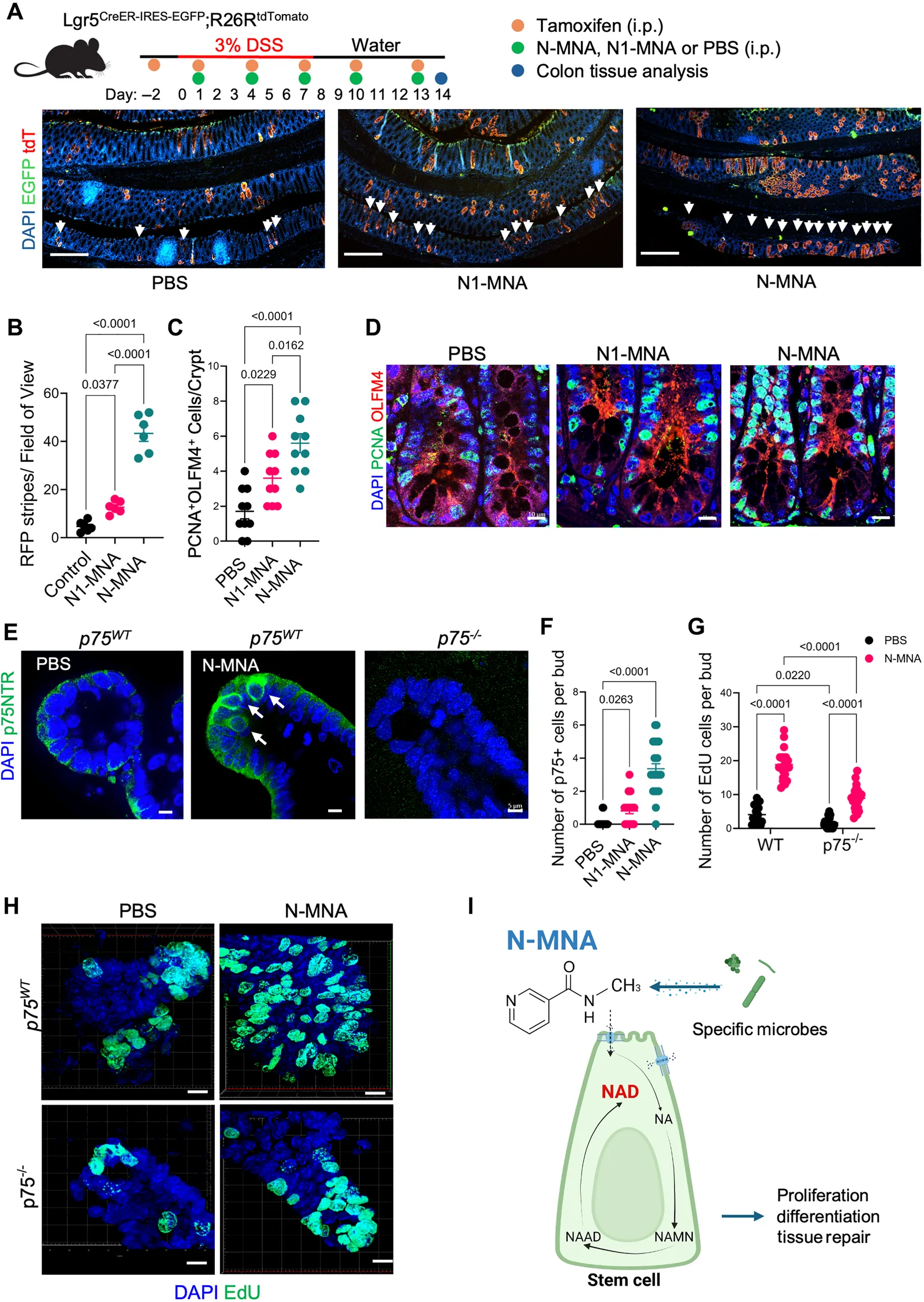
N-MNA drives stem cell renewal for epithelial repair. (**A**) Schematic showing i.p. tamoxifen injections to label intestinal stem cells (ISCs) in *Lgr5^CreER-IRES-EGFP^; R26R^tdTomato^* mice (yellow), i.p. injections of PBS, N1-MNA, or N-MNA (green), and tissue collection (blue). All mice were challenged with 3% DSS in water for 7 days and then switched to regular water for 7 days. Colons were stained for RFP to identify red ISC-driven lineage-tracing stripes. Distal colonic regions containing red stripes are indicated by white arrows. Scale bar, 100 μm. (**B**) Number of RFP-labeled lineage stripes per field of view (n = 6). Ordinary one-way ANOVA with Tukey’s multiple-comparisons test. (**C**) Number of PCNA+ proliferative cells per ileal crypt in mice treated with PBS, N1-MNA, or N-MNA (n = 10). Ordinary one-way ANOVA with Tukey’s multiple-comparisons test. (**D**) Representative immunofluorescence staining for PCNA and the intestinal stem cell marker OLFM4 in ileum from mice treated with PBS, N1-MNA, or N-MNA. Scale bar, 10 μm. (**E**) Immunofluorescence staining for NGFR (p75NTR) in *WT* and *p75^NTR−/−^* enteroids treated with PBS or N-MNA. Arrows indicate cells with high NGFR expression. Scale bar, 5 μm. (**F**) Quantification of NGFR-high cells in enteroid buds shown in (E) (n = 25 buds per group). One-way ANOVA with Tukey’s multiple-comparisons test. (**G**) *WT* and *p75^NTR−/−^* enteroids were treated with PBS or N-MNA for 24 hours and labeled with EdU for 30 min before fixation. Representative EdU staining; scale bar, 5 μm. (**H**) Quantification of EdU^+^ cells per epithelial bud in *WT* and *p75^NTR−/−^* enteroids treated with PBS or N-MNA. One-way ANOVA with Tukey’s multiple-comparisons test. (**I**) Proposed model in which bacteria-derived N-MNA is taken up by intestinal stem cells and incorporated into NAD through the Preiss-Handler pathway, promoting stem cell renewal during tissue repair. Created in BioRender. Han, J. (2026) https://BioRender.com/sflucnp.

To elucidate potential N-MNA downstream molecular effectors relevant to cell proliferation, we re-analyzed the cell-cycle pathway gene set that was uniquely induced by N-NMA (*P* = 1.14e-30) in the Caco2 cell bulk RNA-seq results (fig. S7F). We found that *NGFR* (also known as *p75NTR*), a cell-surface receptor implicated in neuronal cell proliferation and survival (*90*, *91*), showed the greatest, 108-fold, increase by N-MNA (fig. S7, G and H). However, NGFR’s impact on intestinal epithelial proliferation has not been reported. Immunofluorescence staining for p75^NTR^ revealed enhanced expression in a subset of N-MNA-treated WT enteroids (Fig. 7, E and F). No staining was detected in *p75 ^−/−^* mouse enteroids (Fig. 7E). N-MNA-induced enteroid cell proliferation was partially blunted in *p75 ^−/−^* enteroids (Fig. 7, G and H). These results suggested that p75^NTR^ could be one of N-MNA’s regulatory targets that promote cell proliferation.

## DISCUSSION

NAD^+^ is depleted during aging and in various diseases due to reduced biosynthesis and increased consumption in response to DNA damage, oxidative stress, and inflammation (*66*). This imbalance in NAD supply leads to a decline that compromises cellular energy metabolism, redox balance, and tissue homeostasis. The number of clinical trials testing NAD^+^ supplementation to address diverse disorders has grown rapidly. Here, we identified a previously unknown NAD^+^ precursor that is derived from bacteria and differs from the canonical B3 vitamins. Unlike nicotinamide and nicotinic acid, which consume a methyl group from the cellular methyl pool (*7*), N-MNA brings in a methyl group to the cellular methyl pool while replenishing intracellular NAD (Fig. 7I). This offers a novel mechanism that may be beneficial for DNA modification, homocysteine conversion, folate, and methionine metabolism (*92*).

Our cross-species stable isotope tracing studies uncovered an evolutionarily conserved utilization of N-MNA for NAD biosynthesis. Observed conversion of N-MNA to NAD^+^ in *Nampt*-deficient mouse spheroids suggests that this NAD^+^ biosynthetic route is independent of the salvage mechanism in mammalian cells. The NAMPT-dependent salvage pathway dominates mammalian NAD^+^ metabolism. In the absence of NAMPT function, this N-MNA-to-NAD route is of significant scale. We observed a contribution by isotope-labeled N-MNA to one-third of the total cellular NAD in *Nampt*-deficient spheroids after 12 hours of supplementation. Therefore, the N-MNA utilization system may safeguard cellular redox homeostasis under metabolic stress or NAD^+^ depletion.

Blocking Preiss–Handler pathway diminished NAD synthesis in both mouse and human cells, suggesting that N-MNA is likely metabolized by host enzymes into a Preiss–Handler pathway intermediate. The identities of these N-MNA metabolizing enzymes remain unclear, but our experiments indicate multiple routes of initial utilization of this metabolite. In flies, isotope tracing of N-MNA suggested a possible conversion into NAM, which was not evident in mammalian cells. In mouse spheroids and Caco2 cells, N-MNA is possibly converted by multiple enzymes to a Preiss-Handler intermediate, such as NA, to enter the biosynthetic route. The distinct mechanisms in utilizing N-MNA between fly and mammalian cells underscore diversifications in NAD^+^ salvage mechanisms. The difference may reflect the progressive specialization of NAD^+^ metabolism in higher organisms, balancing metabolic flexibility with tighter regulatory control of methylation and redox signaling. The fly and mammalian cells are known to carry different sets of metabolic enzymes for NAD^+^ biosynthesis and metabolism: flies rely on Naam while mammals depend on NAMPT to utilize NAM. Additionally, flies do not have a counterpart of mammalian NNMT that methylates NAM into N1-MNA. An extensive phylogenetic study previously revealed a coevolution and kinetic interplay between NNMT and NAMPT that enables extensive NAD signaling (*93*).

LGG is one of the most consumed probiotic bacteria and is extensively studied for the treatment and prevention of intestinal barrier and inflammatory disorders. We previously reported that LGG modulates the host metabolome, restricts pathobiont expansion, dampens gut inflammatory response, enhances epithelial tight junctions, and regulates Paneth cell biology (*89*, *94*, *95*). LGG is identified here as one of the top producers of N-MNA in our study. Mammalian NNMT makes N1-MNA by methylation of nicotinamide. At the same molar concentration, N-MNA, as a structural isomer of N1-MNA, is more potent in regulating epithelial cell transcriptome, proliferation, and reparative activity. N-MNA is more protective against gut barrier damage in mice and *Drosophila*. N-MNA promotes ISC cell proliferation in fly midgut and mouse colon in response to DSS-induced mucosal damage. ISC-specific *Sirt1* loss only blunted the effect of N-MNA, while enterocyte-specific loss of *Sirt1* in flies diminished all protections by both N- and N1-MNA. These results support that while N-MNA exerts its protection uniquely through ISCs, both N- and N1-MNA can act through enterocytes. The unique ISC-stimulating effect by N-MNA is interesting for the mucosal tissue repair. One of the top transcriptional targets of N-MNA is NGFR, a receptor of neurotrophins, which is known to be accumulated in the epithelial cells during inflammation and promotes regenerative proliferation (*96*). Additionally, LGG has been linked to neuroprotective and stress-modulating effects through gut–brain axis signaling, supporting mood regulation and cognitive resilience in mice (*97*). LGG may synthesize circulating metabolites, such as N-MNA, to mediate its benefits beyond the gastrointestinal tract.

Our stable isotope tracing study confirmed that N1-MNA cannot be utilized for the NAD^+^ biosynthesis. Nevertheless, the two MNAs may functionally converge on Sirtuins, as N1-MNA has been reported to inhibit NNMT. Thus, the supplemented N1-MNA may indirectly increase cellular NAM and NAD^+^ by inhibiting NAD^+^ to NAM flux (*98*). Prior studies also reported that N1-MNA stabilizes SIRT1 protein by inhibiting its ubiquitin-dependent degradation (*99*).

Limitations of our study are noted. First, stool or serum N-MNA may be used as a biomarker for metabolic intervention for specific patients. Although N-MNA was detected in human stool samples, future studies will be required to increase the sample sizes so a stronger linkage of N-MNA with disease states and intervention can be established. Second, the enzymes that metabolize N-MNA remain unknown at this moment, thus future genetic and pharmacological work are required to screen and identify these enzymes. Bacterial comparative and functional genomics will be necessary to reveal the phylogenetic relationship of N-MNA-producing bacterial species and genes. Finally, in addition to its role for NAD^+^ biosynthesis, N-MNA carries a methyl-group into cells likely driving one-carbon metabolism by fueling the methionine cycle, which produces S-adenosylmethionine (SAM) -- the universal methyl donor for hundreds of crucial reactions, including DNA methylation, histone modification, and nucleotide synthesis, vital for gene regulation and cell growth. Indeed, our bulk RNA-Seq data suggested a broad and potent transcriptomic regulation by N-MNA. SIRT1 inhibitor assay did not support a strong regulation on NGFR expression. Thus, the SIRT1 and NGFR pathways are likely independently controlled by N-MNA. Harnessing the therapeutic potential of N-MNA in the future may be beneficial for chronic metabolic and inflammatory diseases in susceptible populations.

## MATERIALS AND METHODS

Reagents used in the study are listed in Table 1.

**Table 1.** Key reagents, resources, suppliers, and corresponding catalog or identifier information used in the experiments.

Chemicals/Antibodies	Company	Catlog #
Nicotinamide (NAM)	Sigma-Aldrich	72340
N1-methylnicotinamide (N1-MNA)	MedChemExpress	HY-1005-24-9
N-methylnicotinamide (N-MNA)	MedChemExpress	HY-124124
^2^H_4_-N1-MNA	A Chemtek	3106-60-3
^2^H_4_-N-MNA	MedChemExpress	2708278–64-0
Dextran sulfate sodium	Thermo Fisher Scientific	0216011080
Sucrose	Thermo Fisher Scientific	BP220–1
Fluorescein sodium salt	Sigma-Aldrich	F6337
Erioglaucine disodium salt	Sigma-Aldrich	861146
anti-SIRT1	Cell Signaling Technology	clone 1F3
anti-GAPDH	Cell Signaling Technology	3683S
anti-RFP	Thermo Fisher Scientific	200–101–379
anti-PHH3	Cell Signaling Technology	9715
anti-GFP	Developmental Studies Hybridoma Bank	clone ID2-S
anti-PCNA	Cell Signaling Technology	2586S
anti-OLFM4	Cell Signaling Technology	39141S
Anti-p75^NTR^	Millipore Sigma	07–476
Click-iT™ EdU Imaging Kit	Thermo Fisher Scientific	C10637
Phosphate Bufered Saline	Thermo Fisher Scientific	MT21040CV
DMEM	Thermo Fisher Scientific	MT10013CV
Fetal bovine Serum	GIBCO	16000044
Primocin	Thermo Fisher Scientific	NC9392943
Penicillin/Streptomycin	Thermo Fisher Scientific	15140122
DMEM F12	Thermo Fisher Scientific	12634010
B-27	Thermo Fisher Scientific	17–504-044
N2	Thermo Fisher Scientific	17–502-048
N-acetylcysteine	R&D	7874/100
EGF	R&D	2028-EG-200
Noggin	Thermo Fisher Scientific	250–38
R-sponding1	R&D	3474-RS-01 M
TGF-β inhibitor (SB-431542)	MedChemExpress	HY-10431G
Rock inhibitor	Sigma-Aldrich	Y0503-1MG
4-OHT	Sigma-Aldrich	H6278–10MG
NAD/NADH Assay Kit	abcam	ab176723
1-[4-(trifluoromethyl)-3-pyridinyl]-ethanone	Sigma	AMBH9884D5B7-100MG
Recombinant human CES2 protein	Creative biomart	CES2-107H
Recombinant Human AADAC protein	Cusabio	CSB-EP001014HU

### Bacterial strains

*R. gnavus* strains ATCC 29149, ATCC 35913, DSM 108212 (H2–28), AGR 2154, and *R. gnavus* E1, B07, J62, G5 isolates were obtained from the American Type Culture Collection (ATCC, USA) or from the DSMZ–German Collection of Microorganisms and Cell Cultures or from Micalis INRAE internal biobank (*100*–*102*). These strains were cultured anaerobically at 37°C in a PLAS-LAB anaerobic chamber (Model 857-OTA) in 14-mL tubes containing 10 mL AnaeroGRO Chopped Meat Glucose Broth (CMB) (Hardy Diagnostics, AG19H). *R. gnavus* isolates (E1, B07, J62, G5) were cultured anaerobically at 37°C in Brain Heart Infusion (BHI) medium (Thermo Fisher, BP9722–2). *L. rhamnosus* GG (ATCC 53103) was cultured in de Man, Rogosa, and Sharpe (MRS) medium (Labviva, K15) under aerobic or anaerobic conditions at 37°C. *Salmonella enterica* serovar *Typhimurium* strain SL1344 and *E. coli* ATCC 10798 were grown in Luria–Bertani (LB) broth (Sigma, 1.10493.0500) supplemented with 100 μg/mL ampicillin at 37°C under anaerobic conditions. *Clostridioides difficile* VPI 10463 (ATCC 43255), *P. acnes* (ATCC6919), and *Citrobacter rodentium* DBS100 (ATCC 51459) were grown anaerobically in BHI broth. *B. breve* (ATCC 15700) was cultured anaerobically in MRS broth +0.05% cysteine-HCl at 37°*C. B. thetaiotaomicron* DSM 2079 and *Muribaculum intestinale* DSM 28989 were grown anaerobically in CMB broth. *A. muciniphila* (ATCC BAA-835) and *E. coli* Nissle 1917 were grown anaerobically in BHI broth, and *Clostridium sporogenes* (ATCC 15579) was cultured anaerobically in Reinforced Clostridial Medium (RCM) broth (Fisher Scientific, DF1808–17-3), all at 37°C. For the collection of spent medium for metabolomic analysis, all cultures were grown to the stationary phase.

### *Drosophila* and husbandry

The following strains were obtained from the Bloomington *Drosophila* Stock Center (BDSC) and their corresponding stock numbers are shown in bracket: *w^1118^* (WT, 3605), *Sirt1^17^* (24857), *Sirt1^2A-7-11^* (8838), *esg > GFP* (*P{GawB}NP5130*, *UAS-GFP/CyO*; derived from 67085 by removing *UAS-Cas9* transgene), *UAS-Sirt1-IR* (*UAS-Sirt1-IR^HMS00484^*; 32481), *mex1-GAL4* (91369), *UAS-Sirt1* (44216), *Naam^102^* (*Naam^MI10214^*; 53231), *Naam^123^* (*Naam^MI12364^*; 58512) (see Table 2).

**Table 2.** *Drosophila melanogaster* strains used in this study, including genotypes, sources, and corresponding stock numbers or identifiers.

Name	Genotype	Vendor	Stock Number
*Wild-type* (*WT*)	*w[1118]*	BDSC	3605
*Sirt1^17^*	*w[1118];Sirt1[17]/SM6a*	BDSC	24857
*Sirt1^2A-7-11^*	*w[1118];Sirt1[2A-7-11]*	BDSC	8838
*esg > GFP*	*P{w[+mW.hs] = GawB}NP5130;P{w[+mC] = UAS-GFP.U}2/CyO;P{y[+t7.7]w[+mC] = UAS-Cas9.P2}attP2*	BDSC	67085
*UAS-Sirt1-IR*	y[1]sc[*]v[1]sev[21];P{y[+t7.7]v[+t1.8] = TRiP.HMS00532}attP2	BDSC	32841
*mex1-GAL4*	*w[1118]; TM2, P{w[+mC] = mex1-GAL4.2.1}12–8/TM6B, Tb[1]*	BDSC	91369
*UAS-Sirt1*	*w[1118]; P{w[+mC] = UASp-Sirt1.Myc}9c*	BDSC	44216
*Naam^MI10214^*	y[1] w[*]; Mi{y[+mDint2] = MIC}Naam[MI10214]/TM3, Sb[1] Ser[1]	BDSC	53231
*Naam^MI12364^*	y[1] w[*]; Mi{y[+mDint2] = MIC}Naam[MI12364]/TM3, Sb[1] Ser[1]	BDSC	58512

To generate *Sirt1* mutant flies, *Sirt1^17^* was crossed with *Sirt1^2^A-7-11*. Stem cell-specific *Sirt1* knockdown lines (*esg > Sirt1-IR*) were generated by crossing *esg > GFP* with *UAS-Sirt1-IR* (32481) and crossing *mex1-GAL4* with *UAS-Sirt1-IR* to obtain enterocyte-specific *Sirt1* knockdown (*mex1 > Sirt1-IR*) flies. For *Sirt1* overexpression, *esg > GFP* was crossed with *UAS-Sirt1-myc* to generate *esg > Sirt1* flies, *mex1-GAL4* was crossed with *UAS-Sirt1* to generate *mex1 > Sirt1* flies, and Naam knockout flies were generated by crossing the *Naam^102^* and *Naam^123^* alleles to produce trans-heterozygous Naam-deficient Drosophila. Experimental crosses were maintained under standard husbandry conditions at 25°C with a 12/12-hour light/dark cycle.

*D. melanogaster* was maintained at 25°C on standard cornmeal–yeast–agar medium (per 3 L: 25.6 g agar, 80.6 g brewer’s yeast, 190.6 g cornmeal, 40.3 g sugar, 16.6 g propionic acid, and 13.3 g tegosept). On the treatment day, adult *Drosophila* were first transferred to empty polypropylene vials containing no food and placed in a 25°C incubator for 3 h. Following starvation, *Drosophila* were moved to treatment vials, each contained a piece of Whatman filter paper saturated with the designated compound solution (vehicle control, 5% DSS, ^2^H_4_-N-MNA, ^2^H_4_-N1-MNA, N-MNA, N1-MNA) at the bottom. After 4 h of treatment at room temperature, *Drosophila* were transferred back onto standard fly medium and maintained at 25°C until collection for downstream assays.

### Mice

Wild type C57BL/6 J mice (Jackson Laboratory, JAX 000664), *Lgr5^EGFP-IRES-CreERT2^* mice (JAX 008875), *Rosa26R^tdTomato^* reporter mice (JAX 007914), and p75^NTR^ knockout (p75^NTR^-KO) mice (JAX 002124) have been reported. Intestinal epithelial cell specific *Nampt* knockout mice (*Nampt^ΔIEC^*) were developed by crossing *Nampt*^fl/fl^ mice (*103*) with Villin-Cre mice (JAX 004586) (*83*). The *Lyz1^3′UTR-IRES-CreER^* mice have been previously described by us (*89*). All mice were maintained on a C57BL/6 background. Animal experiments were approved by the Institutional Animal Care and Use Committee (IACUC, #999900786) at Rutgers New Jersey Medical School. Mice were housed in individually ventilated cages under specific pathogen-free conditions, maintained on a 12-h light/dark cycle, and fed with food and water ad libitum. All experiments were performed on littermates that were randomly divided into treatment groups. At least three independent litters are used for each experimental comparison. Depending on intra-group variations, animal numbers are determined by power analysis to achieve meaningful statistical analysis. Experiments are typically repeated two to six times on different days.

Germ Free (GF) C57BL/6 mice are purchased from Charles River Laboratories and housed Iso-Positive ventilated Tecniplast cage in Newark Gnotobiotic Core Facility at Rutgers New Jersey Medical School. Isolator sterility is monitored on a weekly basis by cultivation-dependent methods for bacterial and mycotic contamination, including aerobic and anaerobic cultures of drinking water and fresh fecal samples from animals housed in each isolator in different media. Strict standard operating procedures are followed for the introduction of materials into isolators.

### Cell lines

Human cell lines Caco-2 BBE (ATCC CRL-2102), HEK293 (ATCC CRL-1573), HeLa (ATCC CCL-2), and HepG2 (ATCC HB-8065) were maintained under standard culture conditions. All cell lines were authenticated by the supplier and routinely tested negative for mycoplasma contamination. Cells were cultured at 37°C in a humidified incubator with 5% CO_2_. Caco-2 BBE cells were maintained in Dulbecco’s Modified Eagle Medium (DMEM; high glucose) supplemented with 20% fetal bovine serum (FBS), 1% Primocin, and 1% penicillin–streptomycin. HEK293 and HeLa cells were cultured in DMEM +10% FBS with 1% penicillin–streptomycin. HepG2 cells were maintained in DMEM (high glucose) or MEM supplemented with 10% FBS, 1% penicillin–streptomycin, and 1% non-essential amino acids, according to ATCC recommendations. Cells were passaged at approximately 70–80% confluence using 0.05% trypsin–EDTA and seeded for downstream assays, including metabolite supplementation, stable isotope tracing, and biochemical analyses. All experiments were performed using cells within 10–17 passages to ensure physiological consistency.

Human iPSC cell line KOLF2.1 J was purchased from Jackson Laboratory (JIPSC001000), and was cultured in mTeSR Plus complete medium (100–0276, Stemcell Technologies) on SyntheMAX (3535, Corning)-coated 6-well plates. Cells were passaged every 3–5 days using ReLeSR (100–0483, Stemcell Technologies). RevitaCell supplement (A26445, Gibco) was added to the culture on the first day after passaging to inhibit Rho-kinase (ROCK). For neural progenitor cell (NPC) differentiation, iPSCs were seeded at 2 × 10^5^ cells/cm2 in a 6-well plate in neural induction medium (08581, Stemcell Technologies) for 21 days and passaged every 7 days. RevitaCell was supplemented on the first day of each passage. After day 21, neural progenitor cells (NPCs) were cultured and expanded in STEMdiff Neural Progenitor Medium (05833; Stemcell Tech), followed by differentiation into human neurons (iNeuron) using forebrain neuron differentiation and maturation media (08600 and 08605, Stemcell Technologies) as described previously (*104*).

### Human stool samples

Human fecal samples were collected at the Center for Comprehensive Treatment of Inflammatory Bowel Disease Regional Hospital No. 2 in Rzeszow, Department of Internal Diseases, University of Rzeszow, Poland, under approval by the local bioethics commission (approval number KE-0254/68/2015). Samples were collected from 20 healthy control patients, 25 ulcerative colitis (UC) patients, and 29 Crohn’s disease (CD) patients. Written informed consent forms were obtained from all study participants. DNA was extracted from stool samples with a QIAamp PowerFecal Pro DNA Kit (QIAGEN, Hilden, Germany) and analyzed by 16S amplicon profiling. Sample processing and data analysis were conducted as described, with the additional pool of samples from UC patients (*105*). For stool calprotectin was quantified using FDA-approved Bühlmann fCAL ELISA kit (BÜHLMANN Laboratories AG, Schönenbuch, Switzerland) using manual extraction protocol according to the manufacturer’s instructions. Calprotectin values were expressed as mg/g of stool sample weight.

### Metabolite extraction and liquid chromatography-mass spectrometry analysis

For metabolite extraction from *Drosophila* tissues, samples were flash-frozen in liquid nitrogen and stored at −80°C until processing. To extract metabolites, samples were homogenized by 1.5 mL pestle (Axygen, PES-15-B-SI) driven by an electrical homogenizer, followed by precipitation in ice-cold methanol of 4 times of the volume. After vortex and incubation on ice for 1 hour, lysates were cleared by centrifugation, and the resulting supernatant was subjected to polar metabolite extraction in acetonitrile/methanol/water (40:40:20, v/v/v). Extraction buffer volume was adjusted as equal of sample volume. Samples were centrifuged at 17,000g for 10 min at 4°C, and the metabolite-containing supernatant was collected. A 150-μL aliquot of each extract was concentrated in a speed vacuum (Eppendorf, Vacufuge plus) and reconstituted in extraction buffer prior to LC–MS analysis.

For bacterial metabolite detection, all bacterial strains were cultured in specific medium and grown to the stationary phase (≥24 hours). Spent media were collected after centrifugation (4000g, 10 min, 4°C) and extracted using the same protocol; uninoculated (blank) media for each bacterial strain were used as control and baseline.

For mouse fecal metabolomics, freshly collected fecal pellets were processed on the designated experimental days. Approximately 30 mg of fecal material per sample was weighed, and metabolites were extracted immediately to preserve metabolic stability. Each milligram of feces was resuspended in 10 μL of ice-cold PBS, homogenized thoroughly using a probe sonicator to disrupt the material, and subjected to protein precipitation with methanol, followed by polar metabolite extraction as described above. Clarified extracts were processed for LC-MS analysis. Fecal metabolite profiling focused primarily on NAD-related metabolites and MNA derivatives. For human stool samples, approximately 50 mg samples were homogenized and extracted using the same extraction buffer and procedure.

For metabolite extraction from Caco2, HEK293, HeLa, HepG2 cells, and ileum-derived organoids or spheroids, fresh samples were processed immediately. Cells or organoids were seeded at defined densities (∼10^6^ cells or ∼500 organoids/spheroids per well) and treated with isotopically labeled or unlabeled metabolites for the indicated time points according to each experimental design. After treatment, samples were subjected to the extraction buffer and procedure described above.

For metabolite extraction from KOLF2.1 J iNeuron cells. Cells or organoids were seeded at defined densities ∼1.5x10^4^ cells per, and treated with isotopically labeled or unlabeled metabolites for the indicated time points according to each experimental design. After treatment, samples were subjected to the extraction buffer and procedure described above.

The LC-MS/MS was performed on a Q Exactive PLUS hybrid quadrupole-orbitrap mass spectrometer coupled to a Vanquish Horizon UHPLC system (Thermo Fisher Scientific, Waltham, MA) with an XBridge BEH Amide column (150 mm × 2.1 mm, 2.5 μm particle size, Waters, Milford, MA). The HILIC separation used a gradient of solvent A (95%:5% H2O:acetonitrile with 20 mM acetic acid, 40 mM ammonium hydroxide, pH 9.4) and solvent B (20%:80% H2O:acetonitrile with 20 mM acetic acid, 40 mM ammonium hydroxide, pH 9.4). The gradient was 0 min, 100% B; 3 min, 100% B; 3.2 min, 90% B; 6.2 min, 90% B; 6.5 min, 80% B; 10.5 min, 80% B; 10.7 min, 70% B; 13.5 min, 70% B; 13.7 min, 45% B; 16 min, 45% B; 16.5 min, 100% B; and 22 min, 100% B (*106*). The flow rate was 300 μL/min. The column temperature was set to 25°C. The autosampler temperature was set to 4°C, and the injection volume was 5 μL. MS scans were obtained in positive ionization mode with a resolution of 70,000 at m/z 200, in addition to an automatic gain control target of 3 × 10^6^ and the m/z scan range of 72 to 1000. Metabolite data was obtained using the El-MAVEN software package (mass accuracy window: 5 ppm). Processed data were exported to GraphPad Prism for statistical analysis and figure generation. For MS/MS validation, spent medium from *B. thetaiotaomicron*, *L. rhamnosus* GG, and *R. gnavus* ATCC 29149 was analyzed to confirm the identity of N-MNA by comparison with authentic chemical standards. The parallel reaction monitoring (PRM) event was set up to collect the parental ions of m/z 137.07 and fragment them with stepped normalized collision energy (NCE) at 10, 35, and 55.

### Stable isotope tracing

For stable isotope tracing in human cell lines, cells were seeded in 6-well plates at a density of 10^5^ cells/well and grew to 80% confluence. Fresh aliquots of ^2^H_4_-N-MNA or ^2^H_4_-N1-MNA were added to the culture medium at a final concentration of 60 μM. Cells were collected at 0 h (before adding the isotope tracers, 15 min, 1 hour, 3 hours, 6 hours, 12 hours, and 24 hours after adding the tracers. Triplicates were used for each time point.

For stable isotope tracing in *Drosophila*, 4–7-day-old adult flies (n = 35 per group) were starved for 4 hours and treated with ^2^H_4_-N-MNA or ^2^H_4_-N1-MNA at a final concentration of 60 μM in the sucrose based solution for 5 hours. Flies were then transferred to dd water for 30 min to clear luminal residues, flash-frozen in liquid nitrogen, and mechanically separated into heads (n = 29) and bodies (n = 33). Each fraction was homogenized and extracted.

For stable isotope tracing in mouse ileal spheroids grown from WT and *NamptΔ^IEC^* mice. Freshly grown spheroids were passaged. Twenty-four hours after passage, ^2^H_4_-N-MNA was added to the medium at a final concentration of 60 μM. Spheroids were collected at 0 (before adding the tracer), 3, and 12 hours after adding the tracer, washed, pelleted at 300g for 5 min at 4°C, homogenized, and extracted for metabolite analysis. Triplicates were used for each time point.

### *Drosophila* survival analysis

For survival assays, 4–7-day-old adult *Drosophila* were collected and distributed into vials containing fewer than 35 flies, with seven independent replicate vials per treatment group, corresponding to 1-day to 7-day treatment durations. Every day before the treatment, flies were starved for 4 h at 25°C. Flies were then transferred to vials containing filter paper saturated with one of the following solutions: 5% sucrose (control), 5% sucrose +5% DSS, 5% sucrose +5% DSS + 100 μM N1-MNA, or 5% sucrose +5% DSS + 100 μM N-MNA. Treatments were administered for 4 hours daily, and flies were returned to the standard food vials after each treatment.

Flies were treated everyday (Day 1 through Day 7). Each vial represented one discrete treatment duration rather than longitudinal tracking of the same cohort. Mortality was recorded for each treatment-duration group (Day 1–Day 7). Mortality counts were used to generate cumulative survival trajectories.

Survival assays were performed across multiple genotypes, including wild-type (*w^1118^*), *Sirt1* mutant (*Sirt1^17^ / Sirt1^2A-7-11^*), *UAS-Sirt1-IR* vs *mex1 > Sirt1-IR* or *esg > Sirt1-IR*, and *UAS-Sirt1-myc* vs *mex1 > Sirt1-myc* or *esg > Sirt1-myc*, to evaluate tissue-specific contributions of *Sirt1* to DSS- or MNA-modulated survival. Kaplan–Meier survival curves were graphed in GraphPad Prism, and survival possibilities were compared using the log-rank (Mantel–Cox) test. Statistical significance was defined as *P* < 0.05.

### *Drosophila* gut permeability assay

Gut barrier integrity in 4-to-7-day-old-adult *Drosophila* was assessed using a dye permeability assay. Prior to treatment every day, flies were starved for 4 hours in 25°C. Flies were then transferred to vials containing filter paper saturated with one of the following solutions: 5% sucrose (control), 5% sucrose +5% DSS, 5% sucrose +5% DSS + 100 μM N1-MNA, or 5% sucrose +5% DSS + 100 μM N-MNA. Treatments were administered daily for 4 hours, after which flies were returned to vials with food medium containing either a blue dye (Erioglaucine disodium salt, EDS) at 2.5% or a fluorescent dye (fluorescein sodium salt, FSS) at 5%.

Flies fed with FSS-containing food were briefly anesthetized with CO_2_ on day 4 and imaged by a dissection microscope mounted with fluorescence LED light source and camera. Flies were then individually transferred into wells of a 96-well black microplate (VWR, Cat# 82050–774) placed on ice. Fluorescence intensity was measured using a plate reader (Promega BioSystems Glomax Multi) at 495 nm excitation. An untreated wild-type control group was included on each plate to obtain the background fluorescence signal. Increased whole-fly fluorescence signal was interpreted as a loss of gut barrier integrity. Statistical comparisons between treatment groups were conducted using one-way or two-way ANOVA, as appropriate, followed by Tukey’s multiple comparison test to determine significance between conditions.

### Mono-colonization of gnotobiotic mice with *R. gnavus* and *L. rhamnosus GG*

*R. gnavus* (ATCC 29149) or *L. rhamnosus* were grown under anaerobic conditions to the stationary phase and pelleted by centrifugation at 1200g for 10 min. Bacterial pellets were resuspended in sterile PBS at a final concentration of 1 × 10^8^ CFU/mL. Adult C57BL/6 J germ-free mice were randomized and grouped, and orally gavaged with 200 μL sterile PBS, 200 μL *L. rhamnosus*, or 200 μL *R. gnavus*. Successful colonization was verified by qPCR using strain-specific primers. Mice were maintained under sterile isolator conditions throughout the experiment. Fecal samples were collected before colonization and 3 and 21 days after colonization, stored at −80°C until metabolomic analysis.

### Antibiotic cocktail treatment in mice

To induce gut dysbiosis by antibiotics, adult SPF mice were administered a broad-spectrum antibiotic (ABX) cocktail through drinking water consisting of ampicillin (1 g/L), neomycin sulfate (1 g/L), metronidazole (1 g/L), and vancomycin (0.5 g/L) for 7 days. The ABX-containing water was freshly replaced every 48 hours to ensure stability. Stool samples were collected before ABX administration and immediately after the 7-day treatment. Fecal pellets were snap-frozen in liquid nitrogen and stored at −80°C until metabolomic analysis.

### DSS colitis induction and intraperitoneal metabolite administration

For metabolite intervention during chemically induced colitis, mice were administered 3% dextran sulfate sodium (DSS) in drinking water for 7 days, followed by a 7-day recovery period on regular water. During the 14-day experimental course, mice received intraperitoneal (i.p.) injections of N-MNA or N1-MNA freshly dissolved in sterile PBS at a dose of 30 mg/kg body weight every three days. Injections were administered. Control animals received an equivalent volume of PBS. Body weight, activity, posture, fur condition, and overall health were monitored daily to assess DSS-induced disease severity.

At the end of the 14-day protocol, mice were euthanized, and tissues were collected for histology or biochemical analyses. Spleens were excised and weighed. The entire colon was dissected, imaged, and measured for length. Colons were then opened longitudinally, rinsed with cold PBS, Swiss-rolled, and fixed in 10% neutral buffered formalin for paraffin embedding and subsequent histological evaluation.

### Lipocalin-2 ELISA

Fresh cecum contents were collected from individual mice, immediately snap-frozen in liquid nitrogen, and stored at −80°C until analysis. For extraction, cecum content samples were weighed and homogenized in PBS containing 0.1% Tween-20 at a ratio of 50–100 mg feces per mL buffer using a mechanical homogenizer. Homogenates were vortexed thoroughly and centrifuged at 10,000g for 10 min at 4°C to remove debris. The supernatant was collected and assayed for lipocalin-2 using a mouse Lipocalin-2/NGAL ELISA kit (R & D, DY1587–05) following the manufacturer’s instructions. Standards and biological samples were run in replicates on the same plate. Absorbance was measured at 450 nm with wavelength using a microplate reader (Promega BioSystems Glomax Multi). LCN2 concentrations were calculated from a standard curve and normalized to fecal content weight.

### Lineage tracing of Lgr5 stem cells in DSS colitis

Adult *Lgr5^EGFP-IRES-CreERT2^; Rosa26R^tdTomato^* reporter mice were intraperitoneally administered with 2 mg tamoxifen dissolved in coin oil to induce CreER mediated recombination in Lgr5 stem cells. Mice were subjected to the 3% DSS colitis induction experimental procedure with N-MNA or N1-MNA administrations described above. Animals were euthanized at the end of recovery phase (14th day) and intestinal tissues were collected, fixed in 10% paraformaldehyde and paraffin-embedded for immunofluorescence staining to visualize the labeled stem cells and their lineage-traced progenies.

### Intestinal organoid and spheroid culture

The typical mouse intestinal organoids were cultured in ENR-supplemented medium as previously described (*107*, *108*). Briefly, ileal crypts were isolated by incubation in cold PBS containing 0.2 mM EDTA with gentle shaking to release epithelial fragments. Crypts were pelleted, resuspended in growth medium, and embedded at a density of 200–300 crypts per 20 μL Matrigel dome (Corning, CLS356231-1EA). Following polymerization, domes were overlaid with complete organoid culture medium composed of advanced DMEM/F12 (Thermo Fisher, 12634010), 1× B-27 (Fisher Scientific, 17504044), 1× N-2 (Fisher Scientific, 17502048), 100 U/mL Primocin (InvivoGen, ant-pm-1), 1.25 mM N-acetylcysteine (Sigma, A9165-25G), 100 ng/ml EGF (R&D, 2028-EG-200), 125 ng/ml recombinant Noggin (ThermoFisher, 250–38–100uG), and 200 ng/ml R-Spondin1 (R&D, 3474-RS-250).

Ileum spheroids were embedded in Matrigel droplets and grown in 50% L-WRN conditioned medium (Advanced DMEM/F12 base) supplemented with ROCK inhibitor (Y-27632) and TGF-β inhibitor (SB-431542). Spheroids were passaged every 2 days by mechanical dissociation and replating into fresh Matrigel. Wild-type and *Nampt*-deficient spheroids (*NamptΔ^IEC^*) were maintained under the above spheroid culture conditions.

For metabolite supplementation experiments, mature organoids or spheroids were incubated with indicated concentrations and duration of NAM, N1-MNA, and N-MNA, along with PBS serving as a control. Samples were subsequently processed for imaging by a conventional brightfield microscope, immunofluorescence staining, or metabolomic profiling.

### Preiss-Handler pathway inhibition assays in spheroids and Caco2 cells

Spheroids were derived from *Nampt*Δ^IEC^ or wild-type ileum, maintained under spheroid-promoting conditions, and passaged every 2 days. To inhibit the Preiss–Handler pathway, spheroids were treated with 1 mM 2-hydroxynicotinic acid (2-HNA), 24 hours after a passage of the spheroids. 2-HNA is an inhibitor of nicotinic acid phosphoribosyltransferase (NAPRT). After 24 hours, spheroids were subjected to stable isotope tracing experiments as described above. All cultures were monitored and imaged for morphological integrity and viability throughout the experiment. For 2-HNA inhibition in Caco2 cells, cells were treated with 1 mM 2-HNA for 24 hours. ^2^H_4_-N-MNA was added to cells. Cell lysates were collected at 0 (control), 12, and 24 hours, for LC-MS analysis. Each condition had 3 biological replicates.

### NAD fluorometric assay

Spheroids derived from wild-type (WT) and Nampt-deficient (*NamptΔ^IEC^*) mice were cultured in L-WRN conditioned medium for 2 days before treatment. Spheroids were then treated by N-MNA or PBS and collected at 0, 3, 6, and 12 hours for NAD quantification. At each time point, spheroids were first washed with ice-cold PBS and lysed in ice-cold PBS containing a protease inhibitor cocktail (Sigma, 11836170001). Lysates were homogenized by brief ultrasound sonication on ice (3 × 5 s pulses with 20–30 s cooling intervals) and clarified by centrifugation at 12,000–16,000g for 10 min at 4°C. An aliquot of 10 μL of each clarified lysate was reserved for total protein quantification using the BCA Protein Assay Kit (Thermo Scientific, #A55864). The remaining lysate was deproteinized using 10-kDa cut-off spin filters, diluted in the kit Extraction Buffer as required, and processed using the Abcam NAD/NADH Fluorometric Assay Kit (ab176723) according to the manufacturer’s instructions.

Equal volumes of lysate were mixed with the NAD/NADH Cycling Mix and Developer in black 96-well plates, and fluorescence was measured using the recommended excitation/emission settings. Total NAD (NAD^+^ + NADH) and NADH concentrations were calculated from standard curves and normalized to total protein. NAD^+^ levels were obtained by subtraction of NADH from total NAD. All samples were processed in parallel to minimize handling variability.

### Bulk RNA-seq analysis

The Caco2 BBE cell line was cultured in DMEM supplemented with 20% fetal bovine serum, 1% pen-strep (Thermo Fisher, 15140–122), and 0.2% Primocin (Thermo Fisher, NC9141815). The cells were grown at 37°C with 5% CO_2_. Cells were seeded at 10^5^ cells/well in 6-well plates and treated with PBS, N1-MNA, or N-MNA for 24 hours. Six biological replicates for each condition were harvested for lysis in TRIzol reagent (Thermo Fisher Scientific, 15596026) and total RNA extraction by the RNeasy Mini Kit (Qiagen, 74104) with on-column DNase digestion (Qiagen, 79254) to remove genomic DNA contamination. RNA concentration and purity were assessed using a NanoDrop spectrophotometer, and RNA integrity was confirmed with an Agilent 2100 Bioanalyzer. Only samples with an RNA integrity number (RIN) > 8.0 were used for library preparation. RNA-sequencing was performed at PrimBio Research Institute (Garnet Valley, PA, USA).

Briefly, Ribo-off rRNA Depletion Kit (Human/Mouse/Rat) (Vazyme, Cat# N406) was used to remove ribosomal RNA from the sample. Briefly, about 1 mg of total RNA was hybridized to rRNA Probe and treated with RNase H to remove the RNA/probe hybrids. Then, DNase I was used to remove the excess DNA probes. RNA sequencing libraries were prepared using NEBNext Ultra II Directional RNA Library Prep Kit for Illumina (NEB#E7760), following the manufacturer’s instructions. Briefly, rRNA-removed RNA from each sample was fragmented at 94°C for 15 min, in the presence of random primers. Then the first strand cDNA was synthesized using NEBNext First Strand Synthesis Enzyme Mix at 42°C for 50 min, followed by second strand synthesis using NEBNext Second Strand Synthesis Enzyme Mix with dUTP mix at 16°C for 60 min. The cDNA generated was purified with 1.8x AMpureXP reagent (Beckmen Coulter #A63882). End Prep was conducted on the purified cDNA using NEBNext Ultra II End Prep Enzyme Mix and ligated to NEBNext Adapter at 20°C for 30 min. Then USER Enzyme was used to digest the lopped adapters. The adapter ligated cDNA library was purified with 0.9x AMpureXP reagent (Beckmen Coulter #A63882), and enriched by PCR reaction with Index primers (NEBNext Multiplex Oligos for Illumina (96 Unique Dual Index Primer Pairs), cat#E6440). The final library was quantified with Agilent 2100 Bioanalyzer High Sensitivity DNA Analysis (cat#5067–4626).

All final libraries were normalized to a concentration of 750 pM and pooled. About 20 mL of pooled libraries was loaded to a P1 flow cell/ 300 cycle cartridges (2x150bp), and then run on a n Illumina NextSeq 2000 sequencer. The raw sequencing data was demultiplexed and processed with Illumina DRAGEN BCL Convert 4.2.7. The fastq files generated were aligned to human hg19 reference sequence. The RNA expression analysis was conducted using StrandNGS software suite (v3.4) using RPKM or DESeq normalization algorithms. Reads Per Kilobase of transcript, per Million mapped reads counts were generated and used for heatmap generation by utilizing the pheatmap package on R.

Bulk RNA-seq datasets were analyzed to identify transcriptional programs regulated by N-MNA and N1-MNA. Differential expression analysis was performed using DESeq2, and genes with an adjusted *P* value (*P*adj <0.05) were considered significantly regulated. All genes were normalized based on the total RPKMs. To define N-MNA or N1-MNA responsive gene sets, we generated two categories: shared upregulated genes, defined as genes significantly upregulated in both the N-MNA vs PBS and N1-MNA vs PBS comparisons; and N-MNA–specific upregulated genes, defined as genes significantly upregulated only in the N-MNA vs PBS comparison.

Each above gene list was analyzed independently using Enrichr (https://maayanlab.cloud/Enrichr/) to identify enriched pathways, transcription factor networks, ontologies, and biological processes. Enrichment scores and adjusted *P*-values were used to determine significance. Odds ratios were calculated from the corresponding 2 × 2 contingency tables to quantify enrichment effect size, and multiple testing correction was applied using the Benjamini–Hochberg procedure. Pathways with adjusted *P* values below the indicated threshold were considered significantly enriched. Enrichr output plots, including bar graphs and combined scores, were exported and compiled for visualization of enriched pathways associated with N-MNA–dependent and shared transcriptional programs.

### Quantitative PCR analysis of NAD biosynthesis genes in WT and *NamptΔ^IEC^* spheroids

Total RNA was isolated from WT and *NamptΔ^IEC^* ileum-derived spheroids using RNAzol RT (Sigma-Aldrich, R4533) according to the manufacturer’s protocol for total RNA isolation. Briefly, spheroids were collected, lysed directly in 600 μL RNAzol, and phase-separated to obtain RNA-enriched aqueous fractions. RNA was precipitated, washed, and resuspended in nuclease-free water. RNA concentration and purity were determined by Nanodrop spectrophotometry, 500 ng of RNA was used for cDNA synthesis.

cDNA was synthesized using Applied Biosystems reverse transcription kit (ThermoFisher, 4374966), and quantitative PCR (qPCR) was performed using SYBR Green master mix on a real-time PCR system (Sigma, S4833). Gene expression analysis focused on enzymes involved in NAD biosynthesis, including Nampt, Naprt, Nmnat1, Nmnat2, Nmnat3, Pnp1, Nadsyn1, and Nrk1/2. Hprt served as the housekeeping gene for normalization.

Primers were designed using NCBI Primer-BLAST to span exon–exon junctions and avoid genomic DNA amplification, and all primer oligonucleotides were synthesized by IDT DNA. Primer sequences used in this study are listed below:

Nampt: TGGGGTGAAGACCTGAGACA (F), TGGCAGCAACTTGTAGCCTT (R)

Naprt: GCAGGACTGTATGCGCTTTCT (F), GAAGCGGCACACCAGGGA (R)

Nmnat1: CTGTGTGGGGCCAATGAGAG (F), TGACAGCGATGTTGTCAGGC (R)

Nmnat2: CCCATCATGACCGAGACCAC (F), CCCTGGCTCTCTCGAACATC (R)

Nmnat3: TAGCCCCACGGTCACTTTTC (F), GCAGTGGCCACCCTGTTTTA (R)

Pnp1: GGCAGATGCTGTTGGCATGAG (F), GAAACCAAAGACACGGAGCC (R)

Nadsyn1: CCAAAGGCAAAGGTGCAAGA (F), GGTGCATTATAGGCATCCCC (R)

Nrk1: GCTCTTGAAGCTTGCTCTGC (F), CCCTCCGTTTGTCACACCAC (R)

Nrk2: CATAGGCATTGGAGGGGTGAC (F), TTAAAGCCGTCCTCTCCGAC (R)

Relative expression was calculated using the ΔΔCt method, and each reaction was performed in technical triplicate.

### Histology and tissue staining

Mouse intestinal tissues were fixed in 10% neutral formalin overnight, transferred to 70% ethanol the next day, and embedded in paraffin at the Histology Core Facility at Rutgers New Jersey Medical School. Afterward, 4 μm thick sections were prepared from the paraffin blocks, rehydrated, and subjected to Hematoxylin and eosin (H&E), immunofluorescent, or immunohistochemistry staining. Sections were deparaffinized in xylene and rehydrated through a graded ethanol series (100%, 95%, 70%) to distilled water. Slides were then incubated in Vector Hematoxylin for nuclear staining followed by a bluing step in alkaline bluing reagent (Vector Laboratories) for 30–60 seconds to enhance nuclear contrast. After rinsing in running tap water, sections were counterstained with Eosin Y for cytoplasmic visualization. Slides were subsequently dehydrated through graded ethanol, cleared in xylene, and mounted with a permanent mounting medium. Stained sections were imaged using a bright-field microscope under identical exposure and illumination settings.

For immunostaining, tissue sections were rehydrated and underwent antigen retrieval using a 0.1 M citrate acid buffer (pH 6.0), with slides immersed in the buffer at sub-boiling temperature for 10 min before being transferred to running water. The slides were then incubated in blocking buffer (PBS containing 0.1% Triton X-100, 2% normal serum, and 2% bovine serum albumin) at room temperature for 1 hr. Slides were then probed with the primary antibodies (SIRT1, 1:400; GFP, 1:50; pHH3, 1:500; RFP, 1:500; PCNA, 1:1000; OLFM4, 1:400) at 4°C overnight. The following day, the slides were washed three times with PBS and incubated with fluorescent dye-conjugated secondary antibodies for 1 h at room temperature. After additional washes and 4′,6-diamidino-2-phenylindole nuclear counterstaining, the slides were air-dried and mounted with Prolong Gold Antifade (Invitrogen). Fluorescent images were captured using a Zeiss LSM980 confocal microscope and analyzed with Image J (https://imagej.net/ij/).

For immunohistochemistry, slides were treated in sub-boiling antigen retrieval buffer (1 μM citric acid, pH 6.0 or 1 μM EDTA, pH 8.0) for 10 min and then immediately transferred into running water. Slides were blocked with PBS buffer containing 0.1% Triton X-100, 2% Bovine Serum Albumin (Sigma A3294-100G), and 2% normal goat serum for 2 hours at room temperature and then incubated with the indicated primary antibodies at 4°C overnight. The next day, the slides were washed with PBS and incubated with biotinylated secondary antibodies for 1 hour at room temperature. Immunohistochemistry was developed by using the ABC kit (Vector Lab, PK-4000), DAB kit (Vector Lab, SK-4100), and imaged by Nikon TE 2000D with NIS Elements D version 4.4.

### Immunocytochemistry and immunofluorescence analysis

Cultured cells or organoids were processed for immunocytochemistry as follows. Caco-2 BBE cells were cultured on collagen-coated glass coverslips. Intestinal enteroids or spheroids were embedded in Matrigel. For EdU labeling, EdU (10 μM) was added to the culture medium 2 hrs before fixation to label the proliferating cells. EdU+ cells were visualized using the Click-iT EdU Imaging Kit (Thermo Fisher, C10338). For immunostaining, cells or organoids were fixed in 4% paraformaldehyde (15–20 min, room temperature) and washed three times in PBS. Samples were permeabilized in 0.2–0.5% Triton X-100 in PBS for 10–20 min, followed by blocking in 5% normal donkey serum or 5% bovine serum albumin in PBS for 30–60 min at room temperature.

Primary antibodies were diluted at indicated concentrations in blocking buffer and incubated with samples for 1 hour at room temperature (SIRT1, 1:400; p75^NTR^, 1:1000). After three PBS washes, fluorophore-conjugated secondary antibodies (Alexa Fluor series, Thermo Fisher) were applied for 60 min at room temperature in the dark. Nuclei were counterstained using DAPI (1 μg/mL). Samples were mounted using Prolong-Gold mounting medium in chambered slides (Cellvis, C8–1.5H-N). Fluorescent images were acquired using a confocal laser scanning microscope. Image analysis and quantification were performed using ImageJ/Fiji, and EdU^+^ or marker-positive cells were quantified from ≥10 independent fields per sample.

### Western blot

Caco-2 BBE cells were cultured in DMEM supplemented with 20% FBS, 1% penicillin-streptomycin, and 0.2% Primocin at 37°C with 5% CO2 and polarized for 21 days. Cells were treated with PBS, N-MNA, NAM or N1-MNA of indicated concentrations. Protein lysates were prepared using radioimmunoprecipitation assay buffer with protease and phosphatase inhibitors, and protein concentrations were determined via the BCA assay kit. Equal protein amounts (20 μg/load) were subjected to SDS-PAGE and transferred to nitrocellulose membranes. Membranes were blocked in 5% nonfat dry milk in Tris-buffered saline with Tween-20 and then incubated overnight at 4°C with primary antibodies. Following secondary antibody incubation and washing, protein bands were detected using enhanced chemiluminescence and imaged. Densitometry analysis of band intensities was performed using ImageJ, normalizing protein expression to either β-actin or GAPDH.

### In vitro enzymatic experimentation for human CES2 or AADAC

Recombinant human CES2 was purchased from Creative Biomart, and recombinant AADAC was purchased from Cusabio. Individual enzymes (2 μg per 100 μL reaction) were incubated with ^2^H_4_-N-MNA (100 μM) in buffer containing 50 mM Tris-HCl (pH 7.5), 0.1% BSA at 37°C for up to 24 hours. Control reactions included reaction buffer alone, enzyme alone, or ^2^H_4_-N-MNA alone. Reactions were terminated by enzyme denaturation at 100°C for 15 min, followed by LC-MS analysis. The metabolomic data is available in MassIVE MSV000102010.

### Quantification of N-MNA in mouse intestinal luminal contents

Luminal samples were first normalized by weight and homogenized in extraction solvent. Deuterium-labeled ^2^H_4_-N-MNA was spiked into each sample as an internal standard of 1 μM before extraction. After centrifugation, the cleared supernatant was analyzed by LC–MS. The endogenous N-MNA concentration was calculated by normalizing the peak area-top value of unlabeled N-MNA to that of ^2^H_4_-N-MNA (1 μM) in the same sample. Final N-MNA concentrations were obtained after normalization to the sample weight.

### Quantification of extracellular ^2^H_3_-NAM

To quantify extracellular ^2^H_3_-NAM generated from ^2^H_4_-N-MNA in Caco2 cells, cells were incubated with ^2^H_4_-N-MNA (60 μM) for 48 hours. Conditioned medium was collected at 0, 12, 24, and 48 hours for LC-MS analysis. The fresh medium (Corning, 10–013-CM) at 0 hr. contains 32.75 μM NAM, which was used as a standard to calculate the extracellular ^2^H_3_-NAM in conditioned medium, based on the ratio of ^2^H_3_-NAM peak area to the unlabeled NAM peak area in fresh DMEM.

### Impurity quantification of ^2^H_4_-HA in tracer

To determine potential ^2^H_4_-NA impurity in ^2^H_4_-N-MNA tracer, fresh Caco2 culture medium was supplemented with 60 μM ^2^H_4_-N-MNA, and spiked in an unlabeled NA at 10 nM, and analyzed by LC–MS. The unlabeled NA peak area resulting from the supplemented NA was used as a standard ot calculate the ^2^H_4_-NA peak area detected in the tracer. Three independent biological replicates were analyzed, and the ^2^H_4_-NA impurity was determined to be 15.62 nM.

### Statistical analysis

Statistical analyses were conducted using GraphPad Prism 10 (https://www.graphpad.com) and Microsoft Excel Office 365. The data were reported as mean ± SEM. Statistical comparisons for two groups were performed by Student’s *t* test, and for multiple groups, either one-way or two-way ANOVA with Tukey’s or Fisher’s LSD test was used. Kaplan–Meier survival curves were used to graph fly survivability. Survival distributions were compared using the log-rank (Mantel–Cox) test. The significant level of enrichment score in the gene set enrichment analysis was computed by an empirical phenotype-based permutation test conducted to account for the complex correlation structure of gene expression patterns. The phenotype labels were permuted, and then the enrichment score of the gene set was recalculated for the permuted data, thus resulting in a null distribution for the enrichment score. The nominal *P* value of the resulting enrichment score was empirically computed relative to this null distribution as previously described (*109*).
