# Supplementary material for: The (amide-)N-methylnicotinamide is a microbe-derived NAD+ precursor that drives gut stem cell renewal
Source: Sci Adv. 2026 Nov 6;12(45):eaef2882. doi: 10.1126/sciadv.aef2882 (PMC13742186; doi:10.1126/sciadv.aef2882)
Supplement: Supplementary file 1 [file sciadv.aef2882_sm.pdf]

Science Advances
AAAS

Supplementary Materials for

The (amide-)N-methylnicotinamide is a microbe-derived NAD^+ precursor that drives gut stem cell renewal

Jiangmeng Han *et al.*

Corresponding author: Ching-On Wong, chingon.wong@rutgers.edu; Xiaoyang Su, xiaoyang.su@rutgers.edu; Nan Gao, ngao@njms.rutgers.edu

Sci. Adv. **12**, eaef2882 (2026)
DOI: 10.1126/sciadv.aef2882

This PDF file includes:

Figs. S1 to S7

Supplementary Figure 1

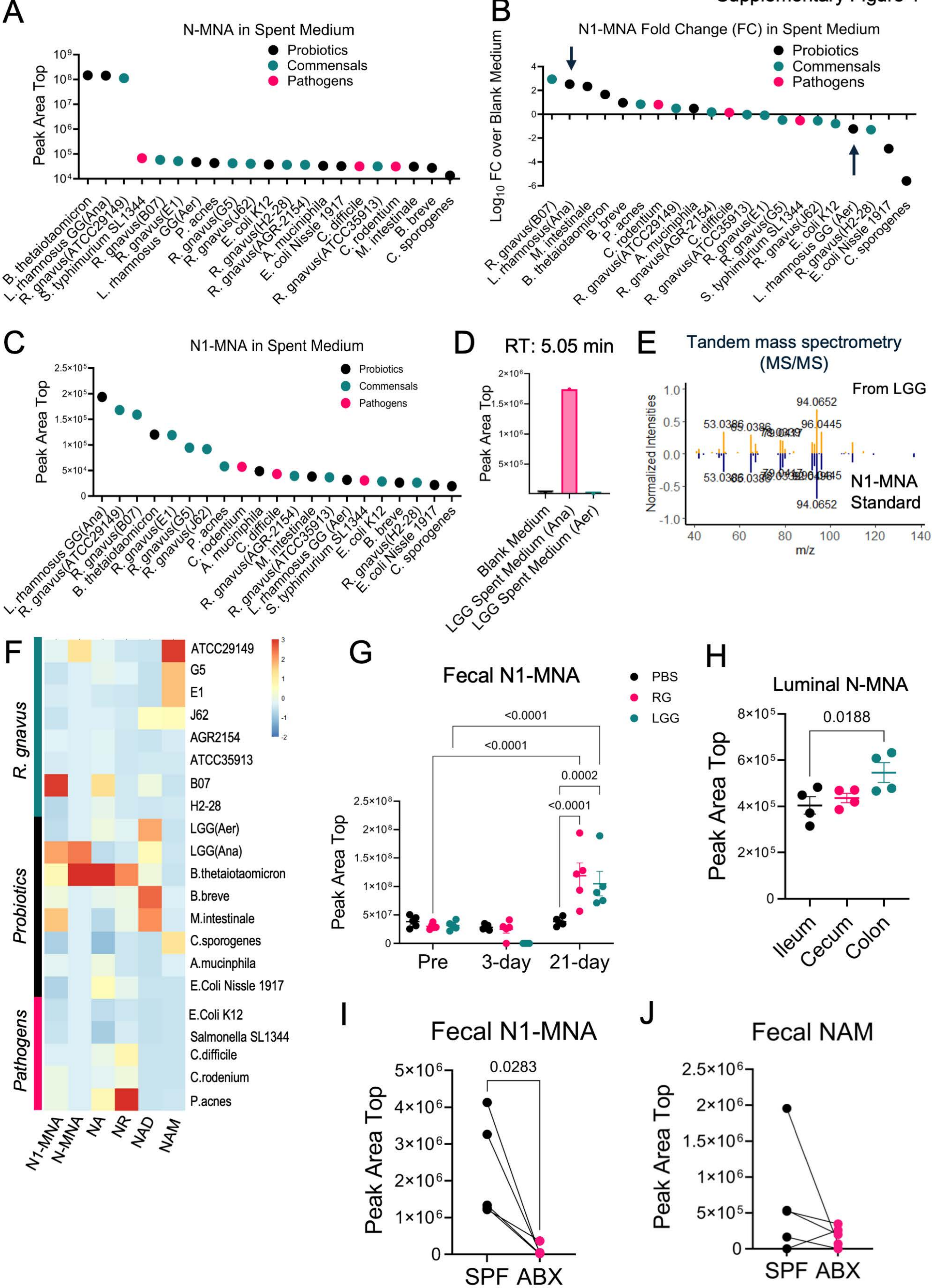

Supplementary Figure 1.

A) Metabolomic absolute value of the peak of N-MNA concentration in 20 species of gut-related bacteria spent medium, compared to their blank culture medium. Gut-related bacteria are separated into three categories: Probiotics (black), Commensal bacteria (green), and Pathogen bacteria (pink).

B) Log_{10} fold-change (FC) of N1-MNA concentration in 20 species of gut-related bacteria spent medium, compare to their blank culture medium. Gut-related bacteria are separated into three categories: Probiotics (black), Commensal bacteria (green), and Pathogen bacteria (pink). Arrow pointed at *LGG* cultured under either aerobic or anaerobic condition.

C) Metabolomic absolute value of the peak of N1-MNA concentration in 20 species of gut-related bacteria spent medium, compared to their blank culture medium. Gut-related bacteria are separated into three categories: Probiotics (black), Commensal bacteria (green), and Pathogen bacteria (pink).

D) N1-MNA peak from LGG spent medium cultured in an anaerobic environment, at a retention time of 5.05 minutes (N1-MNA, pink), compared with N1-MNA in the blank medium (black).

E) MS/MS fraction confirmation of the peak from the LGG spent medium in an anaerobic environment, at a retention time of 5.05 minutes (N1-MNA). Comparison of the sample peak (Yellow), and the N1-MNA standard (Blue).

F) Heat map of all NAD-related metabolites in the 20 species of bacteria spent medium.

G) Germ free mice were inoculated with PBS as control (black), with *R. gnavus* (RG, red) or with *L. rhamnosus GG* (LGG, green). The fecal samples were collected pre-inoculation, 3 and 21 days after mono-colonization. LC-MS was used to detect N1-MNA in individual fecal samples. The peak area top of each sample was normalized against the average fecal N-MNA in pre-treated PBS group and presented as Fold-Change (FC). n=5 mice for each group. Statistical comparison was conducted using two-way ANOVA and Tukey's multiple comparison test.

H) LC-MS quantification of N-MNA in intestinal luminal contents collected from adult SPF mouse ileum, cecum, and colon. The average peak area top of N-MNA in each sample is presented (n=4 mice). Statistical comparison was conducted by one-way ANOVA and Fisher's LSD test for comparison.

I) LC-MS detection of N1-MNA in fecal samples collected from specific pathogen free (SPF) mice that were treated with a cocktail of antibiotics in drinking water for 7 days. The peak area top of each sample was normalized and presented as Fold-Change (FC) against the average of N-MNA levels in pre-treated SPF group. n=5 for each group. Statistical comparison was conducted by a paired student t-test.

J) LC-MS detection of Nicotinamide in fecal samples collected from specific pathogen free (SPF) mice that were treated with a cocktail of antibiotics in drinking water for 7 days. The peak area top of each sample was normalized and presented as Fold-Change (FC) against the average of N-MNA levels in pre-treated SPF group. n=5 for each group. Statistical comparison was conducted by a paired student t-test.

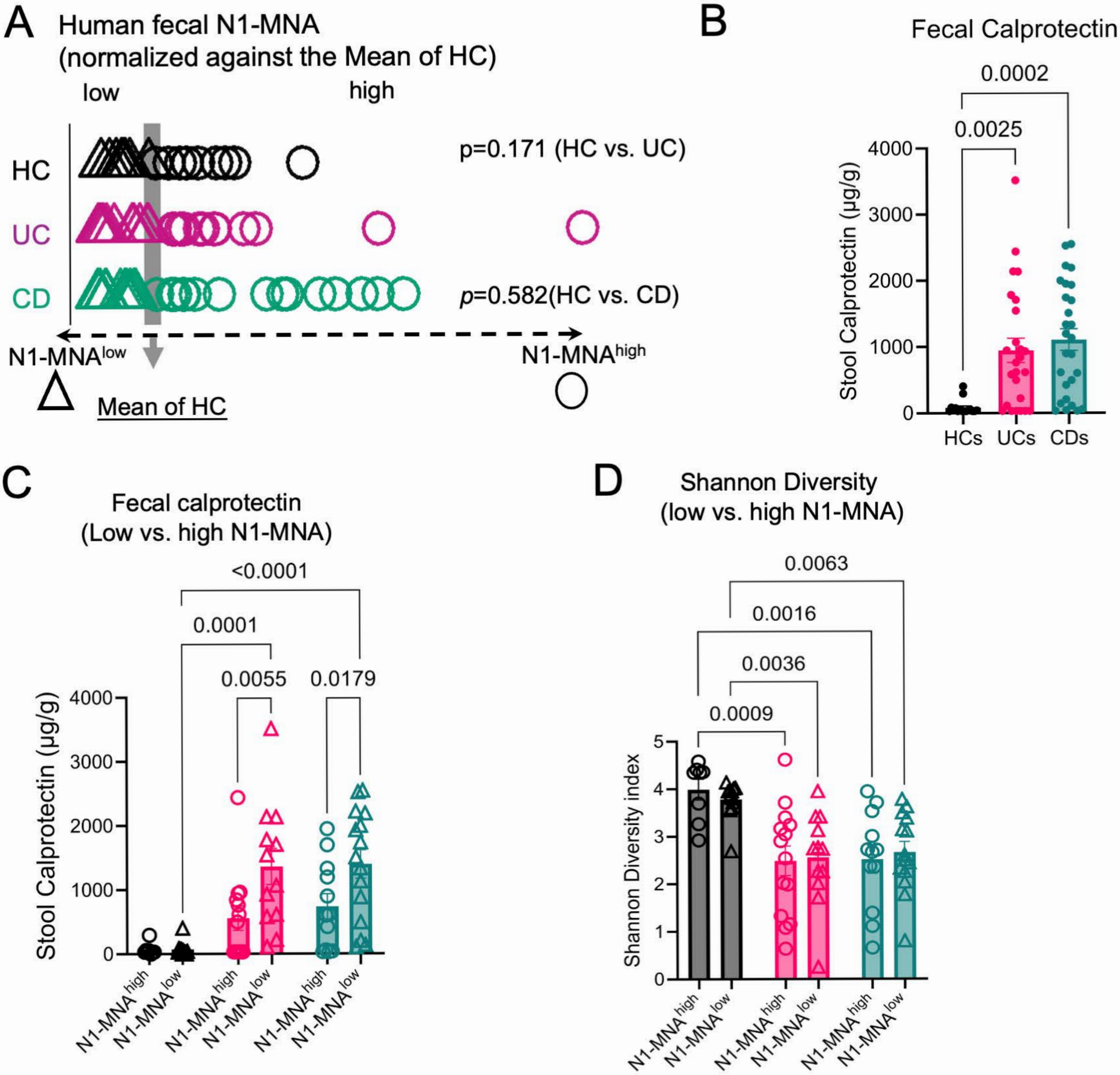
A
Human fecal N1-MNA
(normalized against the Mean of HC)
low
high
HC
UC
CD
p=0.171 (HC vs. UC)
p=0.582(HC vs. CD)
N1-MNAlow
N1-MNAhigh
Mean of HC
B
Fecal Calprotectin
0.0002
0.0025
Stool Calprotectin (µg/g)
HCs
UCs
CDs
C
Fecal calprotectin
(Low vs. high N1-MNA)
<0.0001
0.0001
0.0055
0.0179
Stool Calprotectin (µg/g)
N1-MNA high
N1-MNA low
D
Shannon Diversity
(low vs. high N1-MNA)
0.0063
0.0016
0.0036
0.0009
Shannon Diversity index
N1-MNA high
N1-MNA low

Supplementary Figure 2.

A) Stool N1-MNA levels were measured by LC-MS from 25 ulcerative colitis (UC, pink) and 29 Crohn's disease (CD, green) patients along with 20 healthy control donors (HC, black). Each point represents an individual sample, ordered by increasing N-MNA abundances normalized by the average of healthy controls (indicated by the gray vertical line). Individuals with stool N-MNA above or below HC average are shown in circles or triangles. Statistical analysis was done using one-way ANOVA and Fisher's LSD test for comparison between HC and UC (p = 0.958), between HC and CD (p = 0.0716)

B) Stool calprotectin concentrations in IBD patient fecal samples. IBD patients are divided into three categories: Health (HC, black), Ulcerative Colitis (UC, pink), and Crohn's Disease (CD, green). Statistical analysis was done using one-way ANOVA and Fisher's LSD test for all single comparison

C) Stool calprotectin concentrations were measured from the above groups and plotted as HC, UC and CD cohorts with a comparison between individuals with high or low stool N1-MNA: N1-MNA^{high} and N1-MNA^{low}. Statistical comparison was carried out using a two-way ANOVA and Tukey's multiple comparison test.

D) Metagenomic sequencing of bacterial 16S rRNA was performed from the above groups. Shannon diversity was calculated for individual samples and plotted as HC, UC and CD cohorts with a comparison between individuals with high or low stool N1-MNA: N1-MNA^{high} and N1-MNA^{low}. Statistical comparison was carried out using a two-way ANOVA and Tukey's multiple comparison test.

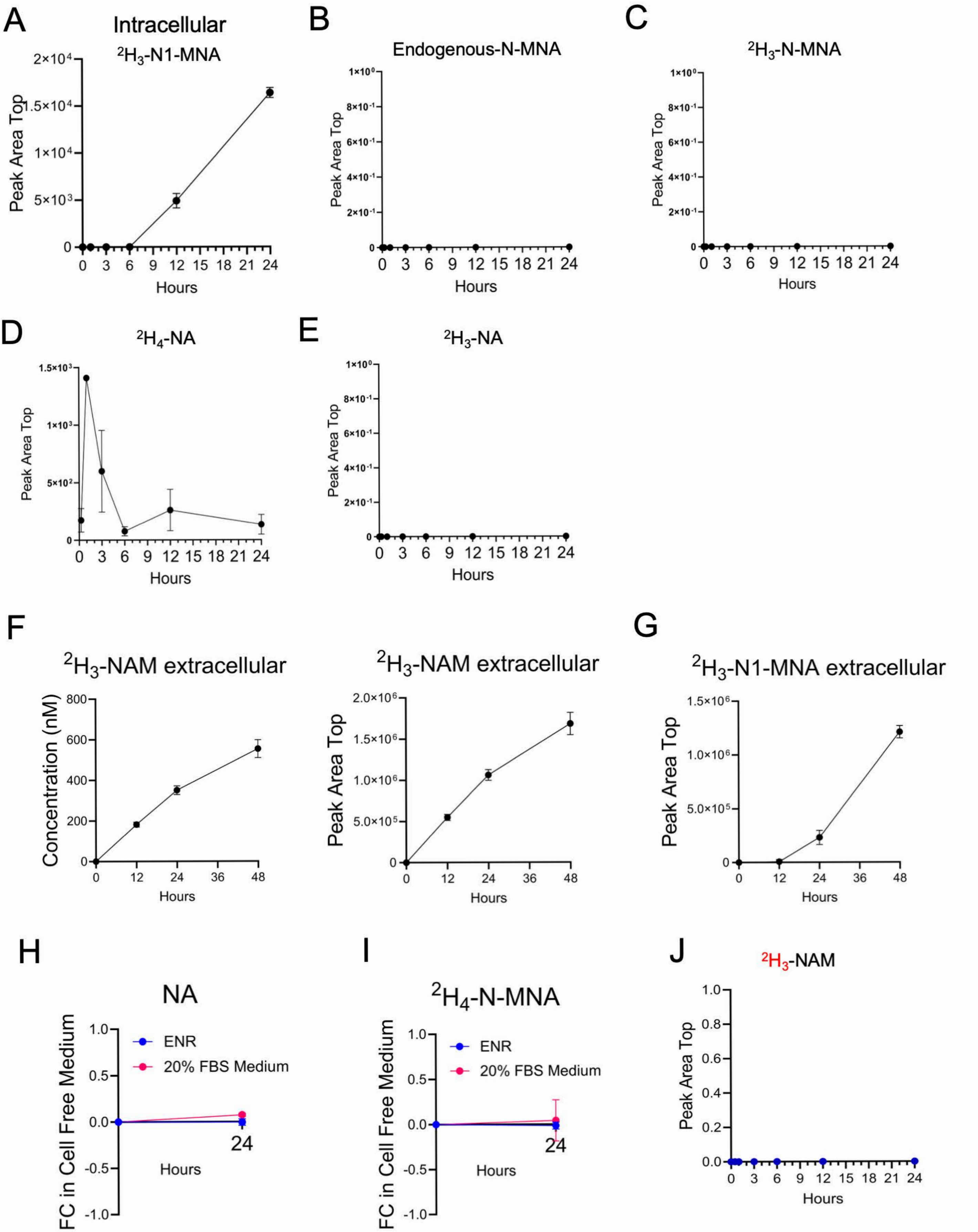
A
Intracellular
$^2H_3$-N1-MNA
Peak Area Top
Hours
B
Endogenous-N-MNA
Peak Area Top
Hours
C
$^2H_3$-N-MNA
Peak Area Top
Hours
D
$^2H_4$-NA
Peak Area Top
Hours
E
$^2H_3$-NA
Peak Area Top
Hours
F
$^2H_3$-NAM extracellular
Concentration (nM)
Hours
$^2H_3$-NAM extracellular
Peak Area Top
Hours
G
$^2H_3$-N1-MNA extracellular
Peak Area Top
Hours
H
NA
ENR
20% FBS Medium
FC in Cell Free Medium
Hours
I
$^2H_4$-N-MNA
ENR
20% FBS Medium
FC in Cell Free Medium
Hours
J
$^2H_3$-NAM
Peak Area Top
Hours

Supplementary Figure 3.

A) $^{2}H_4$-N-MNA was added to confluent Caco2 BBE cells in 6 wells with a final concentration of 60 μM. Before the cells were harvested at 0 hr (pre-treatment), 0.25, 0.5, 1, 3, 6, 12, and 24 hrs, cells were washed extensively with cold PBS to remove unincorporated tracers. Each time point has 3 replicates. Cells were lysed and metabolites were extracted for LC-MS analysis. LC-MS determined the intracellular $^{2}H_3$-N1-MNA in Caco2 BBE cells after addition of $^{2}H_4$-N-MNA in the above time course.

B) LC-MS determined the intracellular unlabeled N-MNA in Caco2 BBE cells after addition of $^{2}H_4$-N-MNA in the above time course.

C) LC-MS determined the intracellular $^{2}H_3$-N-MNA in Caco2 BBE cells after addition of $^{2}H_4$-N-MNA in the above time course.

D) LC-MS determined the intracellular $^{2}H_4$-NA in Caco2 BBE cells after addition of $^{2}H_4$-N-MNA in the above time course.

E) LC-MS determined the intracellular $^{2}H_3$-NA in Caco2 BBE cells after addition of $^{2}H_4$-N-MNA in the above time course.

F) A 48 hrs of pulse tracing of $^{2}H_4$-N-MNA was performed to quantify the extracellular $^{2}H_3$-NAM produced by Caco2 cells. $^{2}H_4$-N-MNA was added to confluent Caco2 cells in a final concentration of 60 μM. Conditioned medium was collected from the same well at 0 hr (before $^{2}H_4$-N-MNA supplementation), 12, 24, and 48 hrs. Each time point has 3 biological replicates. LC-MS determined the extracellular $^{2}H_3$-NAM based on the its peak area ratio to unlabeled NAM (32.75 μM) in the 0 hr medium. At 48 hr, extracellular $^{2}H_3$-NAM was determined to be 554.62 nM.

G) LC-MS determined the extracellular $^{2}H_3$-N1-MNA in Caco2 BBE spent medium 0, 12, 24, and 48 hrs after addition of $^{2}H_4$-N-MNA in above experiment.

H-I) LC-MS determined the stability of $^{2}H_4$-N-MNA and the potential conversion to $^{2}H_4$-NA in cell-free Caco2 culture medium and ENR medium during a 24-hr time course. No change was observed in either metabolite.

A) LC-MS determined the intracellular $^{2}H_3$-NAM in Caco2 cells after addition of $^{2}H_4$-N1-MNA in a time course.

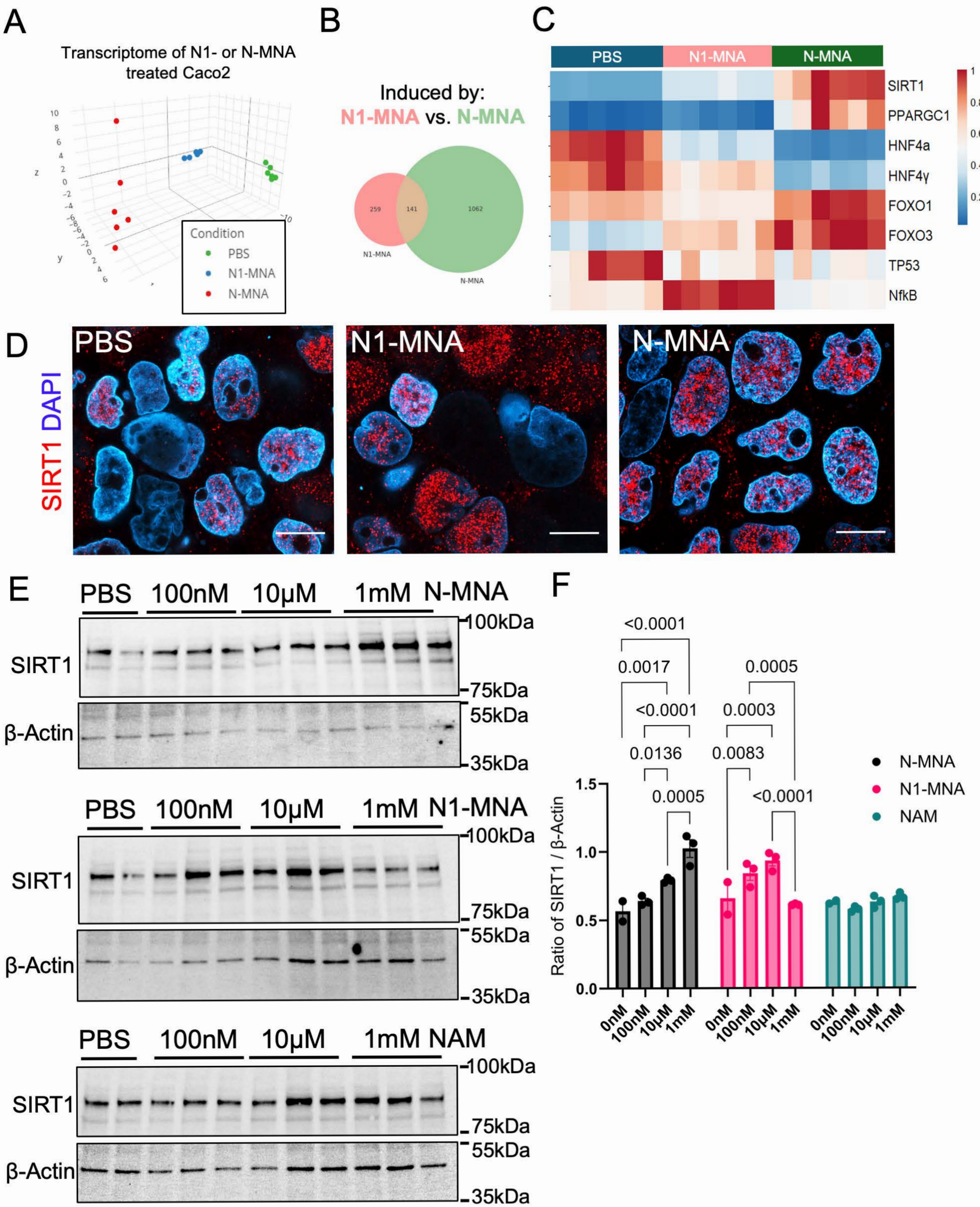
A
Transcriptome of N1- or N-MNA treated Caco2
Condition
PBS
N1-MNA
N-MNA
B
Induced by:
N1-MNA vs. N-MNA
259
141
1062
N1-MNA
N-MNA
C
PBS
N1-MNA
N-MNA
SIRT1
PPARGC1
HNF4a
HNF4γ
FOXO1
FOXO3
TP53
NfkB
D
SIRT1 DAPI
PBS
N1-MNA
N-MNA
E
PBS
100nM
10μM
1mM
N-MNA
SIRT1
β-Actin
100kDa
75kDa
55kDa
35kDa
N1-MNA
NAM
F
Ratio of SIRT1 / β-Actin
<0.0001
0.0017
<0.0001
0.0136
0.0005
0.0005
0.0003
0.0083
<0.0001
N-MNA
N1-MNA
NAM
0nM
100nM
10μM
1mM

Supplementary Figure 4.

A) PCA plot of Caco2 cells treated with PBS (green), N1-MNA (blue), or N-MNA, based on RNA-seq results.
B) Overlapping analysis shows 141 genes shared by N1-MNA and N-MNA-induced genes.
C) Heatmap of SIRT1 and its downstream protein RNA levels.
D) Immunofluorescence staining for SIRT1 (red) in Caco2 cells treated with PBS, N-MNA or N1-MNA. DAPI is shown in blue. Scale bar is 5 μm.
E) Western blot for SIRT1 in the Caco2 cells, which were treated with PBS, NAM, N1-MNA, or N-MNA in various concentrations (0, 100 nM, 10 μM, and 1mM).
F) Western blots were quantified using a two-way ANOVA and Tukey's multiple comparison test.

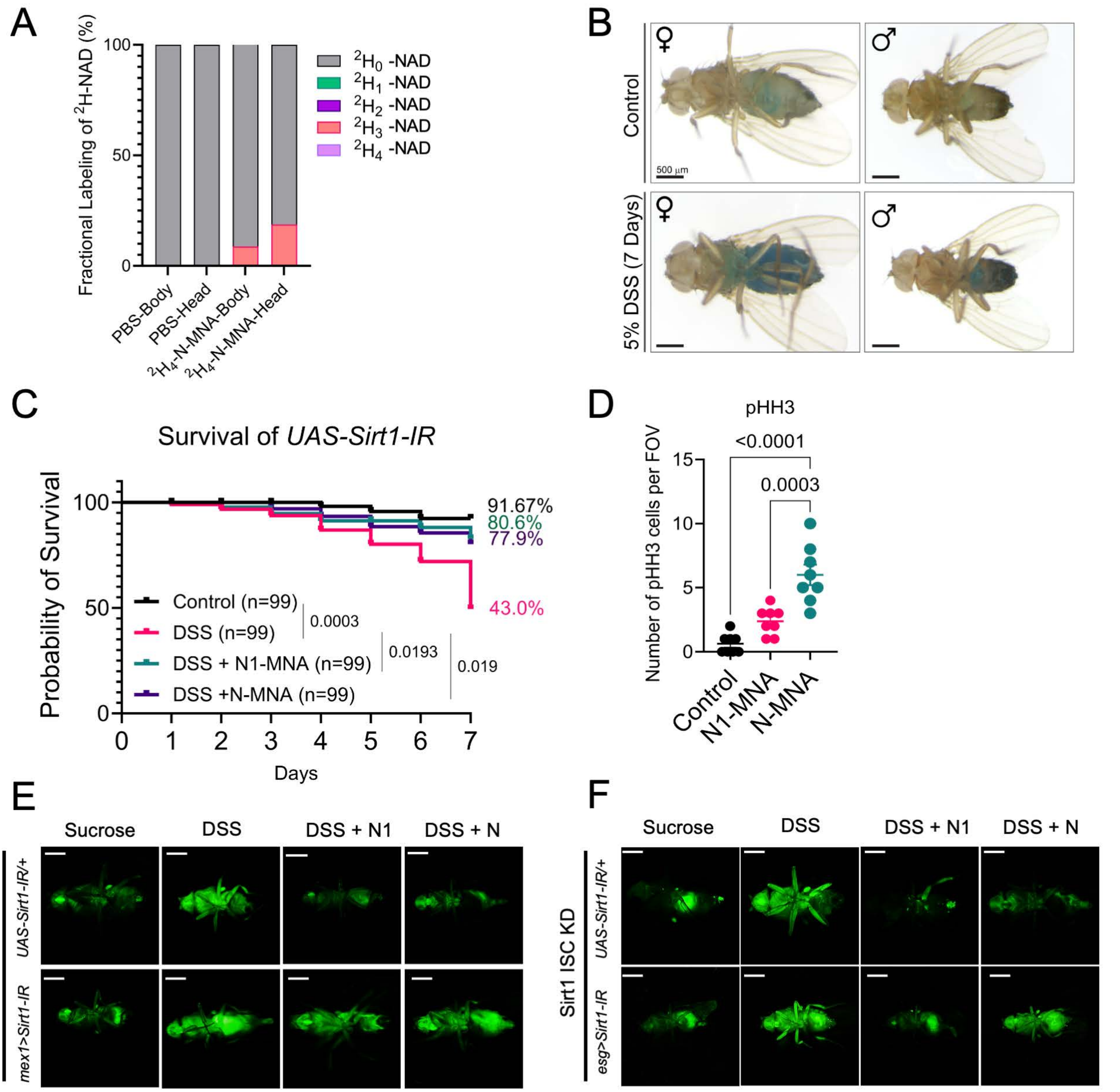
A
Fractional Labeling of ²H-NAD (%)
100
50
0
PBS-Body
PBS-Head
²H4-N-MNA-Body
²H4-N-MNA-Head
²H0 -NAD
²H1 -NAD
²H2 -NAD
²H3 -NAD
²H4 -NAD
B
Control
5% DSS (7 Days)
500 µm
C
Survival of UAS-Sirt1-IR
Probability of Survival
Days
91.67%
80.6%
77.9%
43.0%
Control (n=99)
DSS (n=99)
DSS + N1-MNA (n=99)
DSS +N-MNA (n=99)
0.0003
0.0193
0.019
D
pHH3
Number of pHH3 cells per FOV
<0.0001
0.0003
Control
N1-MNA
N-MNA
E
Sucrose
DSS
DSS + N1
DSS + N
Sirt1 EC KD
UAS-Sirt1-IR/+
mex1>Sirt1-IR
F
Sucrose
DSS
DSS + N1
DSS + N
Sirt1 ISC KD
UAS-Sirt1-IR/+
esg>Sirt1-IR

Supplementary Figure 5.

A) Fractional labeling of NAD ($^2H_0$-$^2H_4$) in *w^{118} Drosophila* fed with or without $^2H_4$-N-MNA after 5.5 hrs. $^2H_0$-NAD (grey),$^2H_1$-NAD (green), $^2H_2$-NAD (purple), $^2H_3$-NAD (pink). $^2H_4$-NAD (indigo). One-way ANOVA and Tukey multiple comparison analysis were performed to compare the differences among various groups

B) Female (left column) and male (right column) Drosophila treated with 5% DSS every day, and fed with EDS in their diet, collected at day 7.

C) Seven-day survival plot of *UAS-SIRT1-IR/+ Drosophila* fed with control, 5% DSS, 5% DSS + N1-MNA, or 5% DSS + N-MNA. n=124 for each group, and the percentage of survival at the endpoint was shown on the graph. Statistical comparison was carried out by using the survival analysis and the individual uncorrected log-rank test.

D) The number of pHH3-labeled cells per field of view in *Drosophila* midgut. Statistical analysis was done using one-way ANOVA and Tukey's multiple comparison test.

E) Adult *mex1>Sirt1-IR Drosophila* and their genetic control were fed with sucrose solution (Control), 5% DSS dissolved in sucrose solution (DSS), 5% DSS sucrose solution containing 100 μM N1-MNA (DSS + N1-MNA) or containing 100 μM N-MNA (DSS + N-MNA), for 4 hrs each day, followed by feeding with a diet containing 2% FSS. Representative images were from *Drosophila* on the 4th day after the first DSS treatment. Scale Bar is 500 μm.

F) Adult *esg*>*Sirt1-IR Drosophila* and their genetic control were fed with sucrose solution (Control), 5% DSS dissolved in sucrose solution (DSS), 5% DSS sucrose solution containing 100 μM N1-MNA (DSS + N1-MNA) or containing 100 μM N-MNA (DSS + N-MNA), for 4 hrs each day followed by feeding with a diet containing 2% FSS. Representative images were from *Drosophila* on the 4th day after the first DSS treatment. Scale Bar is 500 μm.

Supplementary Figure 6

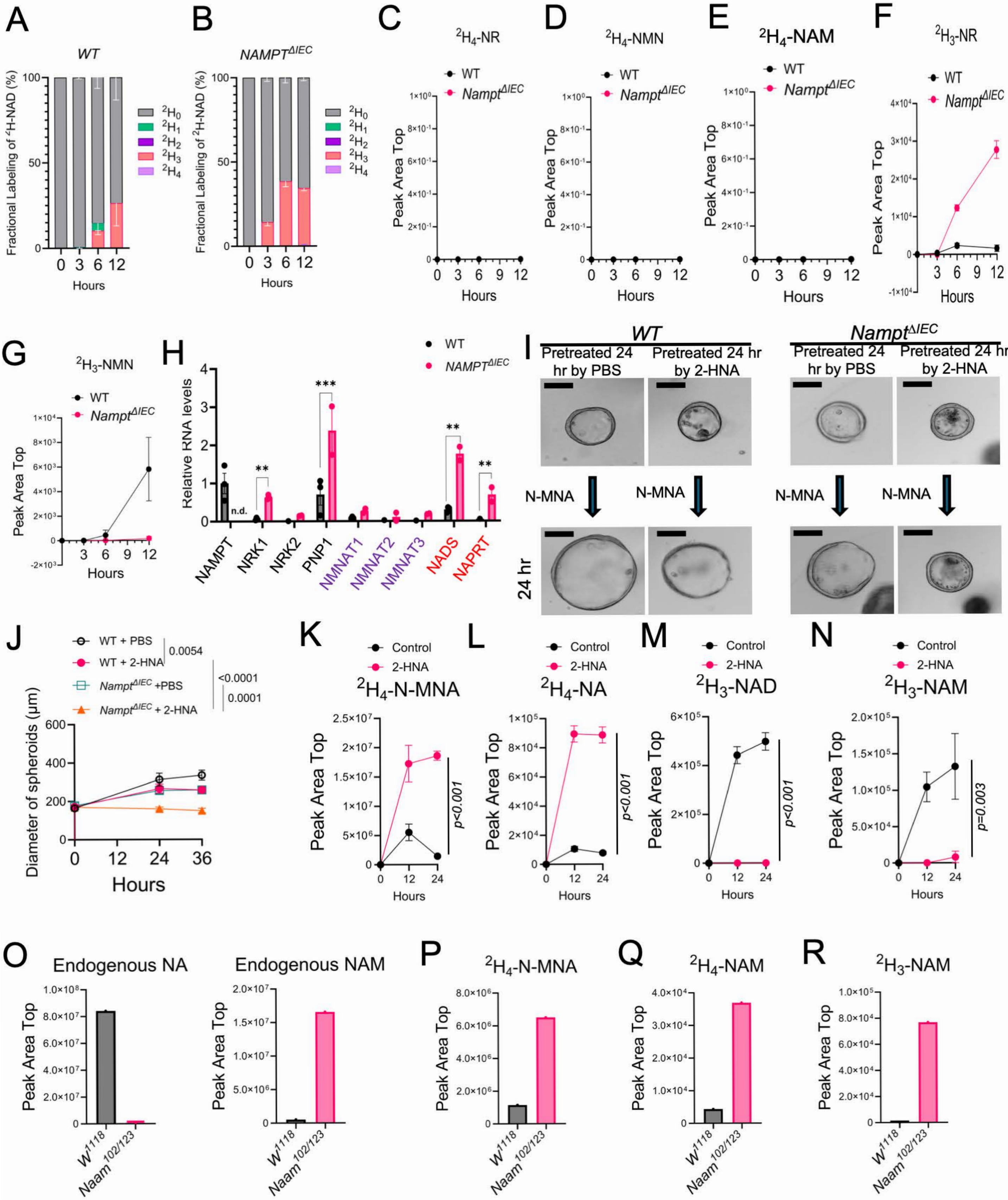

Supplementary Figure 6

A) Fractional labeling of NAD ($^2H_0$-$^2H_4$) in *WT spheroids* supplemented with $^2H_4$-N-MNA and analyzed at 0 hr, 3 hrs, 6 hrs, and 12 hrs. $^2H_0$-NAD (grey),$^2H_1$-NAD (green), $^2H_2$-NAD (purple), $^2H_3$-NAD (pink). $^2H_4$-NAD (indigo). n=3. One-way ANOVA and Tukey multiple comparison analysis were performed to compare the differences among various groups.

B) Fractional labeling of ^2H-NAD ($^2H_0$-$^2H_4$) in *$NAMPT^{\Delta IEC}$ spheroids* treated with the addition of $^2H_4$-N-MNA at 0 hr, 3 hrs, 6 hrs, and 12 hrs. $^2H_0$-NAD (grey),$^2H_1$-NAD (green), $^2H_2$-NAD (purple), $^2H_3$-NAD (pink). $^2H_4$-NAD (indigo). n=3. One-way ANOVA and Tukey multiple comparison analysis were performed to compare the differences among various groups.

C) Stem cell-enriched spheroids were grown from *WT* and *$Nampt^{fl/fl}$; Villin-Cre* (*$Nampt^{\Delta IEC}$*) mice. $^2H_4$-N-MNA was added to the culture at final concentration of 60 μM. After washing with cold PBS to remove unincorporated tracer, the spheroids were collected at 0 (pre-treatment), 3, 6, and 12 hrs. Metabolites were extracted for LC-MS analysis. $^2H_4$-NR in *WT* and *$Nampt^{\Delta IEC}$* spheroids graphed as peak area top at each time point. n=3

D) $^2H_4$-NMN in *WT* and *$Nampt^{\Delta IEC}$* spheroids graphed as peak area top at each time point. n=3.

E) $^2H_4$-NAM in *WT* and *$Nampt^{\Delta IEC}$* spheroids graphed as peak area top at each time point. n=3.

F) $^2H_3$-NR in *WT* and *$Nampt^{\Delta IEC}$* spheroids graphed as peak area top at each time point. n=3.

G) $^2H_3$-NMN in *WT* and *$Nampt^{\Delta IEC}$* spheroids graphed as peak area top at each time point. n=3.

H) qPCR was performed for NAD synthesis pathway enzymes using total RNAs from *WT* and *$Nampt^{\Delta IEC}$* spheroids. Enzymes of the salvage pathway are shown in black; enzymes in the Preiss-Handler pathway are red; and the shared enzymes by both pathways are in purple. n=3 samples for each genotype. Statistical analysis was done using two-way ANOVA and Tukey's multiple comparison test.

I) *WT* and *$Nampt^{\Delta IEC}$* spheroids were treated with or without 2-HNA, in the presence of N-MNA treatment. Representative images were acquired from the same spheroids before and after N-MNA treatment. Scale bar, 100 μm.

J) Spheroid diameters were measured by ImageJ for *WT* and *$Nampt^{\Delta IEC}$* spheroids treated with or without 2-HNA, in the presence or absence of N-MNA (for the last 12 hrs). Two-way ANOVA and Tukey multiple comparison analysis was performed to compare the difference among various treatments.

K) Caco2 cells were treated with 1 mM 2-HNA or PBS for 24 hr. $^2H_4$-N-MNA was added to cells at a final concentration of 60 μM. After washing, cells were collected at 0, 12, and 24 hrs after adding the tracers. LC–MS was performed to measure intracellular $^2H_4$-N-MNA presented as peak area top (n = 3).

L) Intracellular $^2H_4$-NA in above experiment. n=3.

M) Intracellular $^2H_3$-NAD in above experiment. n=3.

N) Intracellular $^2H_3$-NAM in above experiment. n=3.

O) Adult *$Naam^{102/123}$*, and *w^{118}* fly bodies were separated, homogenized, and extracted for LC-MS analysis. Endogenous NAM and NA are plotted. n=36 for each group.

P) Adult *$Naam^{102/123}$* and *w^{118}* flies were fed with $^2H_4$-N-MNA in drinking water for 5 hours and switched to regular water for 30 mins. LC-MS quantitation of $^2H_4$-N-MNA in *Drosophila* bodies. n=36 for each group.

Q) LC-MS quantitation of $^2H_4$-NAM in *Drosophila* bodies in the above experiment. n=36 for each group.

R) LC-MS quantitation of $^2H_3$-NAM in *Drosophila* bodies in the above experiment. n=36 for each group.

Supplementary Figure 7

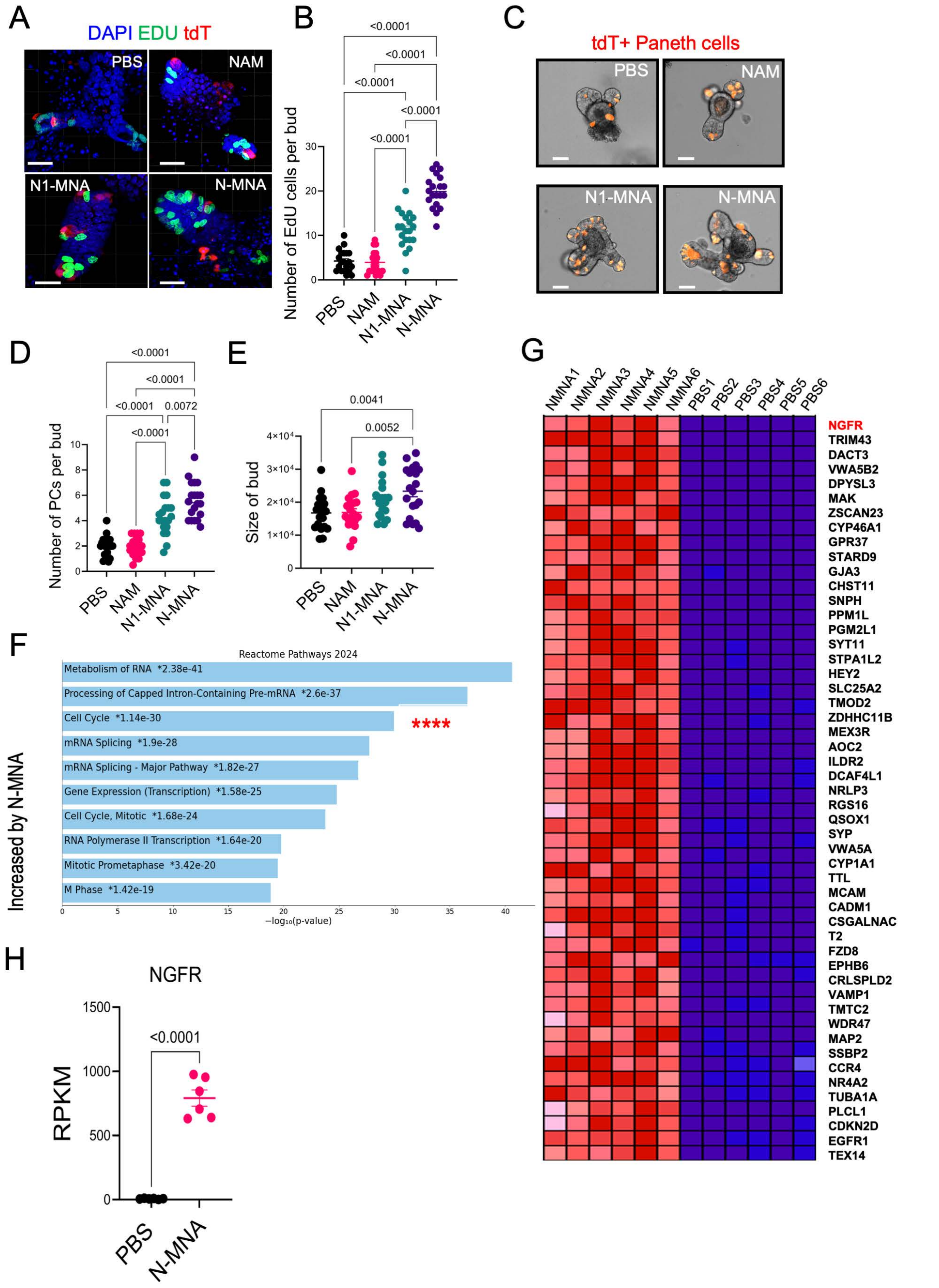

Supplementary Figure 7

A) Mouse enteroids were grown from *Lyz1*$^{3'UTR\text{-}IRES\text{-}CreER}$; *Rosa26R*^{tdTomato} mice. 4-OHT was added to induce tdTomato expression in Paneth cells. On the following day, enteroids were treated with PBS, 100 μM NAM, 100 μM N1-MNA, or 100 μM N-MNA for 24 hrs. Bright field images were overlayed by the red fluorescent images. Scale bar is 10 μm.
B) Ordinary one-way ANOVA and Tukey's multiple comparisons test was used to compare the area (pixel area) of individual epithelial buds of enteroids of different treatments. 20 buds were quantified from each group.
C) Mouse enteroids were grown from *Lyz1*$^{3'UTR\text{-}IRES\text{-}CreER}$;*Rosa26R*^{tdTomato} mice, and were treated with PBS, 100 μM NAM, 100 μM N1-MNA, or 100 μM N-MNA for 24 hr. Enteroids were labeled with EdU for 30 minutes before fixation for staining. Scale bar is 10 μm.
D) Ordinary one-way ANOVA and Tukey's multiple comparisons test was used to compare the number of EdU$^+$ cells in epithelial bud of different treatments. 20 buds were quantified from each group.
E) Number of Paneth cells per bud in each organoid treated with PBS, NAM, N1-MNA, or N-MNA. Statistical analysis was done using one-way ANOVA and Tukey's multiple comparison test.
F) Heat map of the genes from Fig. 7E Cell Cycle (*) pathway. NGFR is the toppest gene labeled in red.
G) Enrichr analysis of N-MNA-induced transcripts, based on Reactome 2024. Pathways related to cell proliferation are indicated by an asterisk. p-values are reported on each bar.
H) RPKM of NGFR from RNA-seq, compared N-MNA-treated Caco2 BBE cells with control groups. Statistical analysis was done using a two-tailed unpaired t-test (n=6).
